# Supplementary material for: Quoted text in the mental healthcare electronic record: an analysis of the distribution and content of single-word quotations
Source: BMJ Open. 2021 Dec 25;11(12):e049249. doi: 10.1136/bmjopen-2021-049249 (PMC8719193; doi:10.1136/bmjopen-2021-049249)
Supplement: Author's manuscript [file bmjopen-2021-049249.draft_revisions.pdf]

BMJ Open

BMJ Open is committed to open peer review. As part of this commitment we make the peer review history of every article we publish publicly available.

When an article is published we post the peer reviewers' comments and the authors' responses online. We also post the versions of the paper that were used during peer review. These are the versions that the peer review comments apply to.

The versions of the paper that follow are the versions that were submitted during the peer review process. They are not the versions of record or the final published versions. They should not be cited or distributed as the published version of this manuscript.

BMJ Open is an open access journal and the full, final, typeset and author-corrected version of record of the manuscript is available on our site with no access controls, subscription charges or pay-per-view fees (http://bmjopen.bmj.com).

If you have any questions on BMJ Open's open peer review process please email info.bmjopen@bmj.com

BMJ Open

Quoted Text in Mental Health Care: Analysis of Distribution and Content

Journal:	*BMJ Open*
Manuscript ID	bmjopen-2021-049249
Article Type:	Original research
Date Submitted by the Author:	19-Jan-2021
Complete List of Authors:	Jayasinghe, Lasantha; King's College London Institute of Psychiatry Psychology and Neuroscience, Department of Psychological Medicine<br>Velupillai, Sumithra; King's College London Institute of Psychiatry Psychology and Neuroscience, Department of Psychological Medicine<br>Stewart, Robert; King's College London Institute of Psychiatry Psychology and Neuroscience, Department of Psychological Medicine; South London and Maudsley NHS Foundation Trust
Keywords:	MENTAL HEALTH, PSYCHIATRY, Health informatics < BIOTECHNOLOGY & BIOINFORMATICS

SCHOLARONE™
Manuscripts

I, the Submitting Author has the right to grant and does grant on behalf of all authors of the Work (as defined in the below author licence), an exclusive licence and/or a non-exclusive licence for contributions from authors who are: i) UK Crown employees; ii) where BMJ has agreed a CC-BY licence shall apply, and/or iii) in accordance with the terms applicable for US Federal Government officers or employees acting as part of their official duties; on a worldwide, perpetual, irrevocable, royalty-free basis to BMJ Publishing Group Ltd ("BMJ") its licensees and where the relevant Journal is co-owned by BMJ to the co-owners of the Journal, to publish the Work in this journal and any other BMJ products and to exploit all rights, as set out in our licence.

The Submitting Author accepts and understands that any supply made under these terms is made by BMJ to the Submitting Author unless you are acting as an employee on behalf of your employer or a postgraduate student of an affiliated institution which is paying any applicable article publishing charge ("APC") for Open Access articles. Where the Submitting Author wishes to make the Work available on an Open Access basis (and intends to pay the relevant APC), the terms of reuse of such Open Access shall be governed by a Creative Commons licence – details of these licences and which Creative Commons licence will apply to this Work are set out in our licence referred to above.

Other than as permitted in any relevant BMJ Author's Self Archiving Policies, I confirm this Work has not been accepted for publication elsewhere, is not being considered for publication elsewhere and does not duplicate material already published. I confirm all authors consent to publication of this Work and authorise the granting of this licence.

For peer review only

Quoted Text in Mental Health Care: Analysis of Distribution and Content

Lasantha Jayasinghe[1], Sumithra Velupillai[1], Robert Stewart[*,1,2]
[1] *Institute of Psychiatry, Psychology and Neuroscience, King's College London, London, UK,*
[2] *South London and Maudsley NHS Foundation Trust, London, UK*

Correspondence to: Lasantha Jayasinghe. lasantha.jayasinghe@kcl.ac.uk

Other Author emails:
Sumithra Velupillai: Sumithra.Velupillai @kcl.ac.uk
Robert Stewart: robert.stewart@kcl.ac.uk

Keywords: electronic health records, mental health, natural language processing, unsupervised learning, k-means clustering

Word count: 3420

For peer review only

ABSTRACT

Objective:

To investigate the distribution and content of quoted text within the electronic health records (EHR) using a previously developed NLP tool to generate a database of quotations.

Design:

Chi-squared and logistic regression were used to assess the profile of patients receiving mental healthcare for whom quotations exist. K-means clustering using pre-trained word embeddings developed on general discharge summaries and psychosis specific mental health records were used to group one-word quotations into semantically similar groups and labelled by human subjective judgement.

Setting:

Electronic health records from a large mental healthcare provider serving a geographic catchment area of 1.3 million residents in South London.

Participants:

For analysis of distribution, 33,499 individuals receiving mental healthcare on the 30th June 2019 in South London and Maudsley (SLaM). For analysis of content, 1,587 unique lemmatized words, appearing a minimum of 20 times on the database of quotations created on the 16th January 2020.

Results:

The strongest individual indicator of quoted text is inpatient care in the preceding 12 months (OR 9.79, 95% CI 7.84 - 12.23). Next highest indicator is ethnicity with those with a black background more likely to have quoted text in comparison to white background (OR 2.20, 95% CI 2.08 - 2.33). Both are attenuated slightly in the adjusted model. Early psychosis intervention word-embeddings subjectively produced categories pertaining to: mental illness, verbs, negative sentiment, people/relationships, mixed sentiment, aggression/violence and negative connotation

Conclusions:

For peer review only - http://bmjopen.bmj.com/site/about/guidelines.xhtml

The findings that inpatients and those from a black ethnic background more commonly have quoted text raise important questions around where clinical attention is focused and whether this may point to any systematic bias. Our study also shows that word embeddings trained on early psychosis intervention records are useful in categorizing even small subsets of the clinical records represented by one-word quotations.

For peer review only

For peer review only - http://bmjopen.bmj.com/site/about/guidelines.xhtml

ARTICLE SUMMARY

Strengths and limitations of this study

- A large sample size (n= 33,499) for logistic regression of all patients receiving mental healthcare on the 30th June 2019 was used in this study and comparisons made between characteristics of groups with/without a quotation.
- Pre-trained word-embeddings used to categorise one-word quotations based on a large electronic mental health record corpus of around 23 million words each.
- 27% (9,118) of the data for those with quotations on the 30th June 2019 contained variables with missing values and therefore were not included in the adjusted logistic regression analysis
- Investigator subjective judgement is used to determine the category label of clusters and consequently the optimum number of clusters.

For peer review only - http://bmjopen.bmj.com/site/about/guidelines.xhtml

INTRODUCTION

Mental health electronic health records (EHRs) routinely capture a wealth of unstructured information detailing a patient's clinical journey including symptoms, observed behaviours, contextual factors, assessments, interventions and outcomes within the free-text fields of case notes and correspondence (1). The majority of studies using this information have focused on these clinical constructs (2). However the EHR is also a narrative account written from the perspective of healthcare professionals (3). Within this account, clinicians often add exact quotes from patient testimony and other parties, for example as evidence for their diagnosis or other decisions (4,5). This has the potential to give insight into the patient voice, albeit as secondary information filtered through the lens of the clinician, reflecting both the focus of the encounter and the individual clinician's reporting style.

Previous research has focussed on the amount of quoted text in the EHR. For example, a study by our group investigated the frequency of quoted text in the EHR at time periods close to a suicide attempt (6). Other studies, rather than examining quoted speech directly, have looked at instances of 'referencing', where the source of text is assigned to a third party using the 'he/she says' construct. In one study there was a greater relative frequency in third-person pronoun use in a group of veterans who had died from suicide, in contrast to a comparison service user group (7), and another study found an increase in referencing when doctors communicated negative news to patients (8).

To our knowledge, the distribution of quoted text within the EHR has received little or no investigation. It is unknown which patients are more likely to have quoted text in their EHR, or if there are variations amongst different demographic or diagnostic groups. Therefore, building on our previous work, the first objective of this study was to ascertain the distribution of quoted text within

For peer review only - http://bmjopen.bmj.com/site/about/guidelines.xhtml

patients receiving secondary mental healthcare. Additionally, no investigation has been undertaken into the content of the quoted text within the EHR. For example, it is unknown whether quotes predominantly relate to psychopathological terms that clinicians have been trained to note down, or whether they cover other indications of patient experience outside medical terminology. Therefore, the second objective of this study was to ascertain the content of these quotations. One way of investigating the content of large volumes of text is by employing natural language processing (NLP) methods. NLP has increasingly been used to extract clinically relevant information such as symptoms and medication from EHRs (9). Most NLP studies involve a corpus of annotated text. As part of the work to investigate quoted text frequencies in relation to self-harm, we developed an algorithm to identify and extract these entities from a large volume of mental healthcare text fields (6). However, as the resulting database of quotations represented a large quantity of unknown data, we decided to use unsupervised methods to classify the quotations. One way of representing words is via word embeddings, where semantically similar words have similar numerical vector representations (10). These vectors are generated by applying machine learning models over specific text corpora. Previous studies on the same mental healthcare platform as our data have generated word embeddings to identify terminology around general symptoms of mental illness (11) and more specifically psychosis (12). Focusing on the one-word quotations, we sought to explore the extent to which these pre-trained embeddings might be used to classify the content of the quotations identified.

For peer review only - http://bmjopen.bmj.com/site/about/guidelines.xhtml

METHODS

Study sample

The South London and Maudsley NHS Foundation Trust (SLaM) provides comprehensive, near-monopoly mental healthcare services to a geographic catchment of around 1.3m residents in four boroughs of south London, as well as some national specialist services. The mental health records used in this study were assembled using SLaM's Clinical Record Interactive Search (CRIS) platform, which currently accesses mental healthcare records for over 500,000 patients, rendering them de-identified and accessible for research use (13). CRIS, as a data resource for secondary analysis, has IRB approval from the Oxford Research Ethics Committee C (reference 18/SC/0372).

The distribution of individuals with quoted text

The first objective of the study was to describe the distribution of those with quoted text at the database creation date, 16th January 2020, on which there were 365,555 total active patients in SLaM from which quotations would potentially arise. The date of the first instance of quoted text for a patient was used as the index date to determine the variable values. Additionally, for comparison purposes we extracted the same variables for all active SLaM patients on a particular index date, 30th June 2019 and compared the people with or without quoted text to see if there were any differences.

Variables

All variables were derived from structured text fields at index date. The basic demographic variables were gender, ethnicity and age. For analytical purposes, we summarized ethnicity into five groups, as follows: White European (British, Irish, Any other white background), Black (African, White and Black African, Caribbean, White and Black Caribbean, Any other black background), Asian (Bangladeshi, Indian, Pakistani, Chinese, Any other Asian background, White and Asian), Other (Any

For peer review only - http://bmjopen.bmj.com/site/about/guidelines.xhtml

other mixed background, Any other ethnic group) and Missing. Other variables included were: the Index of Multiple Deprivation Score, most recent primary diagnosis, whether any inpatient care was received in the preceding 12 months with a binary Yes/No outcome, the number of community face to face contacts in the preceding 12 months and the year of first patient referral to SLaM.

The Index of Multiple Deprivation (IMD) Score (14) represents the socioeconomic status of neighborhoods in the UK by combining various economic, social and housing indicators. It is based on the 2011 national census data and calculated from the patient's most recent address at index date. The IMD score was assigned to tertiles (0 <= x <= 20, 20 < x <= 30, 30 < x <= 93), with lower groups indicating greater deprivation. The most recent primary diagnosis was determined from the ICD10 code assigned to the patient at index date. In this analysis, the groups were represented by the first letter and/or digit of the ICD10 code, resulting in the following categories: F0x, F1x, F2x, F3x, F4x, F5x, F6x, F7x, F8x, F9x, Zx, Any other letter x and Not recorded.

The content of quoted text

The second objective of our study was to investigate the content of quoted text. Previous work by our team involved the development of a natural language processing (NLP) tool to identify text occurring within quotation marks in the EHR using regular expression matching on a sample of patients hospitalised for a suicide attempt (6). The details of the application have been previously described (6), but in summary this algorithm yielded a precision of 0.92, recall of 0.93 against a manually annotated gold standard. As one-word quotations were the largest proportion by word count, these 199,384 instances (27% of the total) were chosen as the focus for this particular investigation.

For peer review only - http://bmjopen.bmj.com/site/about/guidelines.xhtml

Statistical analysis

Data and statistical analysis were performed using standard Python (version 3.6.8) libraries. To analyse the distribution of individuals with quoted text, first the characteristics for all individuals with quoted text at the database creation date of 16th January 2020 were calculated. Where an individual had more than one instance of quoted text, the date of the first quotation was used for variable extraction.

Second, in the sample of patients receiving mental healthcare on 30th June 2019, chi-squared tests were used to test the associations between each individual variable and the presence of quoted text. Chi-squared tests were calculated with missing values excluded and for ordinal variables (age group, index of multiple deprivation, number of face to face community contacts, year of first SLaM referral) as a linear trend with one degree of freedom. Then logistic regression was used to analyse whether individual variables increased the likelihood of the presence of quotations and whether this was attenuated when all variables were incorporated in a single model. The logistic regression analyses were conducted using complete cases only.

To analyse the content of quoted text, first the one-word quotations (199,384) were extracted from the quotations database, lower-cased and lemmatized (converted to its base form) using the NLTK package, WordNetLemmatizer (15) . This resulted in 25,873 unique lemmatized words. Of these, the most frequent quotations, classified as those appearing at least 20 times, were compiled into a list for further analysis, giving 1,587 words. These were then mapped into vector format via the word embeddings and used in the clustering algorithm. We used the pre-trained word embeddings generated by Viani and colleagues (12) trained specifically on CRIS records pertaining to 1) general discharge summaries (23.6m words) reflecting all mental health disorder records (not restricted to

For peer review only - http://bmjopen.bmj.com/site/about/guidelines.xhtml

psychosis) and 2) early psychosis intervention services (23.3 m words) across all mental health services. These were trained using a gensim (16) implementation of Word2Vec with the Continuous Bag of Words (CBOW) model. We felt this was a suitable approach as these embeddings have been specifically trained on CRIS records, so are more likely to reflect the semantic similarity of words in the context specific to our mental health platform. For example, words such as 'hyper' are used more loosely in general terminology, but have specific meanings in the clinical context.

The idea behind clustering methodology is to use an unsupervised algorithm to group together semantically similar words as represented by their vector forms. K-means was chosen as it has high accuracy and speed with large datasets and provides good data segmentation (17). The implementation of k-means used was Python's *sklearn* library, which by default runs the algorithm ten times with different centroid seeds to minimize the impact of clusters forming around local minima. Several methods are available to select the optimum number of clusters. We initially sought to use the 'elbow method' to determine the number of clusters by plotting the inertia for 1 to 20 clusters. However, as no obvious 'elbow' was apparent, we opted for silhouette analysis. Under this method the higher the average silhouette score for each cluster, the better the representation of the data. Therefore, in statistical terms, the optimum number of clusters is represented by the highest average silhouette score over a range of possible values. This was determined by plotting the average silhouette scores for 1 to 20 clusters and examining the highest values. However, as our objective was to classify the content of one-word quotations in a way that added meaning to the groups, it was necessary to use subjective judgement to assess whether the optimum number of clusters provided distinct meaningful groups. To assess the usefulness of the k-means clustering algorithm in assigning semantically similar words to similar groups, the investigator examined the 20 most frequent words in each cluster and using subjective judgement assigned a descriptive label for each cluster. If at least one cluster could be given a homogeneous label, then the process was

For peer review only - http://bmjopen.bmj.com/site/about/guidelines.xhtml

complete and this number of clusters was deemed optimum. Otherwise, the number of clusters with the next highest average silhouette score was assessed in a similar way, and so on until it was possible for the investigator assess at least one of the clusters as a homogenous group.

Patient involvement

We did not directly incorporate patient and public involvement (PPI) into this particular study, but the SLaM BRC Case Register used in the analysis was developed with extensive PPI and is overseen by a committee that includes service-user representatives.

RESULTS

The previously developed tool (6) to identify quoted text from a sample of CRIS records was run over the whole CRIS database, which contained 365,555 records on the 16th January 2020. After removal of blank quotes and those representing 'html' tag data, 728,505 quotations were identified from CRIS, relating to 53,476 individuals. The quotations were further categorised in terms of word count. The mean number of words in a quotation was 9, median 3, standard deviation 25 and range 1-309, indicating a wide distribution in the size of quotations with a positive skew. Details for the volumes of quotations by word length are described in Table 1.

Characteristics of patients with at least one instance of quoted text are displayed in Table 2 and comparisons of patients with/without quoted text on the index date are presented in Table 3. Quoted text was more common in male patients, in those from black compared to white ethnic groups and those living in more deprived neighbourhoods. In terms of clinical variables, quoted text was more common in those with schizophrenia, schizotypal and delusional disorders (F2x), those receiving inpatient care, and those with higher numbers of community face to face contacts in the preceding 12 months.

For peer review only - http://bmjopen.bmj.com/site/about/guidelines.xhtml

The unadjusted and adjusted logistic regression results are presented in Table 4. The presence of any inpatient care in the preceding 12 months is the strongest individual indicator of quoted text, with those receiving care nearly ten times more likely to have quoted text than those without. In terms of ethnicity, black individuals are approximately twice as likely as white Europeans to have instances of quoted text, although this is attenuated by the presence of other variables in the adjusted model. In comparison to the reference category, F2x (Schizophrenia, schizotypal and delusional disorders), other primary diagnoses are very unlikely to produce instances of quoted text. Additionally, gender, age group and Index of Multiple Deprivation have very little effect on the presence of quoted text, in the adjusted model.

Cluster Analyses

The optimum number of clusters suggested by silhouette analysis (see Figure 1) for the discharge summary word embeddings was two. This yielded two clusters, which appeared to distinguish between a group referring to sentiment (negative and positive) and a miscellaneous group. As the investigator observed that both groups appeared to contain mixed rather than distinct categories, the next highest silhouette score was examined and this yielded four clusters, which are displayed in Table 5a. This shows that group 0 is miscellaneous with no obvious descriptive category label, whilst the other groups appear to contain words related to mental illness, sentiment and verbs. The optimum number of clusters using the early intervention word embeddings was nine (see Figure 2), as shown in Table 5b. These groups appeared to contain more clearly differentiable categories, relating to mental illness, verbs (2 groups), negative sentiment, people/relationships, mixed sentiment, aggression/violence and negative connotation.

For peer review only - http://bmjopen.bmj.com/site/about/guidelines.xhtml

For peer review only

13/ 42

DISCUSSION

To our knowledge, this was the first study to describe the distribution of quoted text and the content of one-word length quotations from a large clinical record database, the size of which adds strength to our findings. In a sample of SLaM patients at census date 30th June 2019, those with any inpatient care in the preceding 12 months are most likely to have quoted text in the clinical record, even after adjusting for other variables. Ethnicity is the next most pertinent factor, with quoted text appearing more commonly for those with a black ethnic background, but this is attenuated slightly in the adjusted model. Individuals with schizophrenia, schizotypal and delusional disorders (F2x) are much more likely to have quoted text than with any other primary diagnosis, although primary diagnosis in general has little effect in the adjusted model. This study also found that one-word quotations were clustered into more distinctive categories using the early intervention word embeddings in comparison to those generated from discharge summaries. This resulted in nine groups which could be subjectively labelled as follows: mental illness, verbs (2 groups), negative sentiment, people/relationships, mixed sentiment, aggression/violence and negative connotation.

Our findings of any inpatient care in the preceding 12 months being the strongest indicator of quoted text may be due to those hospitalised receiving more frequent clinical observation than outpatients, leading to a greater number of clinical notes from which quotations may arise. Further, there may be a greater focus on quoting text as evidence for decision making and medical defence practice (4,5), given inpatients are more likely to be suffering from the most severe mental health conditions. On a similar basis, a greater frequency of quoted text from those of black ethnicity may be explained by higher levels of psychosis being present in this group in comparison to white ethnic backgrounds (18–20) and consequently these individuals may receive a greater clinical focus.

For peer review only - http://bmjopen.bmj.com/site/about/guidelines.xhtml

The finding that the early psychosis intervention word-embeddings produce more distinct categories in the data makes sense in the context of around 44% of all quotations in CRIS arising from patients with a recent primary diagnosis of F1x - Mental and behavioural disorders due to psychoactive substance use, F2x - Schizophrenia, schizotypal and delusional disorders, or F3x - Mood [affective] disorders, as all these have psychosis as a possible symptom. Therefore, word-embeddings trained on records most similar to those from which the quotations are derived are likely to produce the best results in the clustering process. It is interesting to note that aside from categories related to mental illness and sentiment, this study has uncovered other more unexpected areas where clinicians may quote their patients, in terms of aggression/violence, people/relationships and verbs, indicating an emphasis on the circumstances of a patient's experience rather than purely symptomatology.

This study has several strengths. First it examines a novel area by focussing on the distribution and content of quotations within the EHR rather than the full record, giving insight into information clinicians may quote beyond clinical terminology. Furthermore, the large sample sizes for analysis of both distribution and content add significance to the findings. Additionally, the word-embeddings used to represent the one-word quotations have been trained on millions of words which are highly relevant since they have been derived specifically from mental health records on the same platform.

The findings of our study need to be taken with several limitations in mind. One limitation of our study is that groups referenced may refer to a heterogeneous group of people, for example in terms of ethnicity. Another limitation is that 27% of the sample data were incomplete cases and therefore were not included in the adjusted logistic regression analysis. Another consideration is the data for logistic regression is examined at one point in time, so unknown confounders may be present in the data, such as previous service use for a different mental health disorder. In terms of investigating the

For peer review only - http://bmjopen.bmj.com/site/about/guidelines.xhtml

content of quoted text, one key limitation is that the labelling of groups found by clustering is subjective and based on the assessment of the researcher. Another key limitation is that what is found in the text is dependent on what the clinician notes down, this will be subject to training and individual preferences and biases. Additionally, attribution of the speaker is not determined by the algorithm but the majority were from patients (6). Further, as we chose to investigate one-word quotations as a first step, the meaning derived from words in terms of clustering is limited without context. Therefore, further studies should examine longer strings of quotations to gauge a better understanding of content. Additionally, further studies could use contextual word-vector representations. Under this methodology, words are assigned vector representations based on the surrounding contextual words, to give a better idea of how a specific word is used in a particular context.

In conclusion, despite limitations, this is an important study as the first of its kind to investigate the profile of patients and the areas of patient experience that are highlighted in quoted speech within the clinical record. The successful creation of a database across all CRIS to identify quoted speech is a first step in making this data available for future studies. The findings that inpatients and those from a black ethnic background more commonly have quoted text raise important questions around where clinical attention is focused and whether this may point to any systematic bias. Our study also shows that word embeddings trained on early psychosis intervention records are useful in categorizing small subsets of the clinical records represented by one-word quotations.

For peer review only - http://bmjopen.bmj.com/site/about/guidelines.xhtml

REFERENCES

1. Perera G, Broadbent M, Callard F, Chang CK, Downs J, Dutta R, et al. Cohort profile of the South London and Maudsley NHS Foundation Trust Biomedical Research Centre (SLaM BRC) Case Register: Current status and recent enhancement of an Electronic Mental Health Record-derived data resource. BMJ Open. 2016 Mar 1;6(3):e008721.

2. Velupillai S, Hadlaczky G, Baca-Garcia E, Gorrell GM, Werbeloff N, Nguyen D, et al. Risk Assessment Tools and Data-Driven Approaches for Predicting and Preventing Suicidal Behavior. Front psychiatry [Internet]. 2019 [cited 2019 Jun 19];10:36. Available from: http://www.ncbi.nlm.nih.gov/pubmed/30814958

3. Aaslestad P. The patient as text: the role of the narrator in psychiatric notes, 1890-1990. CRC Press; 2016.

4. GOV.UK. Assessing and managing risk in mental health services - GOV.UK [Internet]. 2009 [cited 2019 Oct 9]. Available from: https://www.gov.uk/government/publications/assessing-and-managing-risk-in-mental-health-services

5. Roth, Lorraine S. Writing progress notes: 10 dos and don'ts [Internet]. MDedge Psychiatry. 2005 [cited 2019 Oct 9]. p. 4(2):63-66. Available from: https://www.mdedge.com/psychiatry/article/59861/writing-progress-notes-10-dos-and-donts

6. Jayasinghe L, Bittar A, Dutta R, Stewart R. Clinician-recalled quoted speech in electronic health records and risk of suicide attempt: A case-crossover study. BMJ Open. 2020 Apr 22;10(4):e036186.

7. Leonard Westgate C, Shiner B, Thompson P, Watts B V. Evaluation of Veterans' Suicide Risk With the Use of Linguistic Detection Methods. Psychiatr Serv [Internet]. 2015 Oct 15 [cited 2019 Jun 5];66(10):1051–6. Available from: http://psychiatryonline.org/doi/10.1176/appi.ps.201400283

For peer review only - http://bmjopen.bmj.com/site/about/guidelines.xhtml

8. Van DeMieroop D. The quotative ‘he/she says’ in interpreted doctor–patient interaction. Interpreting [Internet]. 2012 Jan 1 [cited 2019 Jun 19];14(1):92–117. Available from: http://www.jbe-platform.com/content/journals/10.1075/intp.14.1.05mie

9. Wang Y, Wang L, Rastegar-Mojarad M, Moon S, Shen F, Afzal N, et al. Clinical information extraction applications: A literature review. Vol. 77, Journal of Biomedical Informatics. Academic Press Inc.; 2018. p. 34–49.

10. Mikolov T, Chen K, Corrado G, Dean J. Efficient estimation of word representations in vector space. In: 1st International Conference on Learning Representations, ICLR 2013 - Workshop Track Proceedings. International Conference on Learning Representations, ICLR; 2013.

11. Stewart R, Jackson R, Patel R, Velupillai S, Gkotsis G, Hoyle D. Knowledge discovery for Deep Phenotyping serious mental illness from Electronic Mental Health records [version 2; referees: 2 approved]. F1000Research. 2018;7.

12. Viani N, Patel R, Stewart R, Velupillai S. Generating positive psychosis symptom keywords from electronic health records. In: Lecture Notes in Computer Science (including subseries Lecture Notes in Artificial Intelligence and Lecture Notes in Bioinformatics). Springer Verlag; 2019. p. 298–303.

13. Perera G, Broadbent M, Callard F, Chang C-K, Downs J, Dutta R, et al. Cohort profile of the South London and Maudsley NHS Foundation Trust Biomedical Research Centre (SLaM BRC) Case Register: current status and recent enhancement of an Electronic Mental Health Record-derived data resource. BMJ Open [Internet]. 2016 Mar 1 [cited 2019 Jun 19];6(3):e008721. Available from: http://www.ncbi.nlm.nih.gov/pubmed/26932138

14. Gov. English indices of deprivation 2019 - GOV.UK [Internet]. [cited 2020 Nov 16]. Available from: https://www.gov.uk/government/statistics/english-indices-of-deprivation-2019

15. NLTK. nltk.stem.wordnet — NLTK 3.5 documentation [Internet]. [cited 2021 Jan 6]. Available from: https://www.nltk.org/_modules/nltk/stem/wordnet.html

For peer review only

For peer review only - http://bmjopen.bmj.com/site/about/guidelines.xhtml

16. Gensim. gensim · PyPI [Internet]. [cited 2021 Jan 6]. Available from: https://pypi.org/project/gensim/

17. Rodriguez MZ, Comin CH, Casanova D, Bruno OM, Amancio DR, Costa L da F, et al. Clustering algorithms: A comparative approach. PLoS One [Internet]. 2019 Jan 1 [cited 2020 Aug 3];14(1):e0210236. Available from: https://doi.org/10.1371/journal.pone.0210236

18. Veling W. Ethnic minority position and risk for psychotic disorders. Curr Opin Psychiatry [Internet]. 2013 Mar [cited 2020 Dec 2];26(2):166–71. Available from: http://journals.lww.com/00001504-201303000-00007

19. Coid JW, Kirkbride JB, Barker D, Cowden F, Stamps R, Yang M, et al. Raised incidence rates of all psychoses among migrant groups: Findings from the east london first episode psychosis study. Arch Gen Psychiatry [Internet]. 2008 Nov 3 [cited 2020 Dec 2];65(11):1250–8. Available from: https://jamanetwork.com/journals/jamapsychiatry/fullarticle/482871

20. Kirkbride JB, Hameed Y, Ioannidis K, Ankireddypalli G, Crane CM, Nasir M, et al. Ethnic Minority Status, Age-at-Immigration and Psychosis Risk in Rural Environments: Evidence From the SEPEA Study. Schizophr Bull [Internet]. 2017 Oct 21 [cited 2020 Dec 2];43(6):1251–61. Available from: http://academic.oup.com/schizophreniabulletin/article/43/6/1251/3829518

For peer review only

For peer review only - http://bmjopen.bmj.com/site/about/guidelines.xhtml

TABLES AND FIGURES

Table 1. The word length of quotations in the database

Word length	N	% Total
0	1694	0.23
1	199,384	27.37
2	148,394	20.37
3	86,476	11.87
4	58,993	8.1
5	42,482	5.83
6	30,791	4.23
7	22,616	3.1
8	18,066	2.48
9	13,581	1.86
10 - 14	37,566	5.17
15 - 19	15,936	2.19

For peer review only

For peer review only - http://bmjopen.bmj.com/site/about/guidelines.xhtml

20 - 24	8,205	1.13
25 - 29	5,347	0.73
30 - 34	3,854	0.53
35 - 39	3,036	0.41
40 - 44	2,487	0.35
45 - 49	2,233	0.3
50 - 54	1,951	0.26
55 - 74	6,106	0.84
75 - 99	5,129	0.7
100 - 149	6,713	0.69
150 - 199	4,028	0.68
200 +	3,437	0.68

For peer review only

For peer review only - http://bmjopen.bmj.com/site/about/guidelines.xhtml

Table 2. Characteristics of patients receiving mental healthcare who have at least one quotation on the index date (16th January 2020)

	All patients on the Quotations database*	
	N	% Total
Gender		
Female	26420	49.4%
Male	27035	50.6%
Unknown	21	0.0%
Ethnicity		
White European	31435	58.8%
Black	14155	26.5%
Asian	2592	4.8%
Other	3456	6.5%

For peer review only

For peer review only - http://bmjopen.bmj.com/site/about/guidelines.xhtml

Unknown	1838	3.4%
Age group		
1-15	2463	4.6%
16-25	10363	19.4%
26-35	7417	13.9%
36-45	8484	15.9%
46-55	9198	17.2%
56-65	5942	11.1%
66-75	2976	5.6%
76-85	2751	5.1%
+86	3872	7.2%
Unknown	10	0.0%
Index of Multiple Deprivation		
0 <= x <= 20 - (Most deprived)	15386	28.8%
20 < x <= 30	17026	31.8%

For peer review only

For peer review only - http://bmjopen.bmj.com/site/about/guidelines.xhtml

30 < x <= 93 - (Least deprived)	19323	36.1%
Unknown	1741	3.3%
Most Recent Primary Diagnosis via ICD10 code		
F0x - Organic, including symptomatic, mental disorders	4853	9.1%
F1x - Mental and behavioural disorders due to psychoactive substance use	6065	11.3%
F2x - Schizophrenia, schizotypal and delusional disorders	8773	16.4%
F3x - Mood [affective] disorders	8773	16.4%
F4x - Neurotic, stress-related and somatoform disorders	6248	11.7%
F5x - Behavioural syndromes associated with physiological disturbances and physical factors	1958	3.7%
F6x - Disorders of adult personality and behaviour	2548	4.8%
F7x - Mental retardation	1145	2.1%
F8x - Disorders of psychological developmental	2037	3.8%
F9x - Behavioural and emotional disorders with onset usually occurring in childhood and adolescence	3578	6.7%
Zx - Unspecified	768	1.4%
Any other letter x	376	0.7%

For peer review only

For peer review only - http://bmjopen.bmj.com/site/about/guidelines.xhtml

Not recorded	6354	11.9%
Any inpatient care in the preceding 12 months		
No	52986	99.1%
Yes	490	0.9%
Number of face to face community contacts in the preceding 12 months		
0	40518	75.8%
1-7	5666	10.6%
8-14	2521	4.7%
15-21	1848	3.5%
22-28	1162	2.2%
29-42	1067	2.0%
+43	694	1.3%
Year of first SLAM referral		

For peer review only - http://bmjopen.bmj.com/site/about/guidelines.xhtml

1918 - 2014	50339	94.1%
2015 - 2020	2580	4.8%
Unknown	557	1.0%

*Where a patient had more than one quotation data has been derived from the date of their earliest record

For peer review only - http://bmjopen.bmj.com/site/about/guidelines.xhtml

Table 3. Characteristics of patients receiving mental healthcare on the index date (30th June 2019, n= 33,499) and comparison of those with/without at least one instance of quoted text

	All Patients Active on SLAM at 30th June 2019	Patients with quotations Active on SLAM at 30th June 2019		
	N	N	% All SLAM Active Patients by Row Total	Chi-Square tests of independence[+]
Gender				
Female	15992	4039	25.3%	$\chi^2(1) = 57.81$
Male	17459	5056	29.0%	$p < 0.001$
Missing	48	7	14.6%	
Ethnicity				

For peer review only - http://bmjopen.bmj.com/site/about/guidelines.xhtml

For peer review only

White European	14560	4263	29.3%	$\chi^2(3) = 822.63$
Black	7752	3694	47.7%	$p < 0.001$
Asian	1440	476	33.1%	
Other	1864	488	26.2%	
Missing	7883	181	2.3%	
Age group**				
1-15	6850	669	9.8%	$\chi^2(1) = 28{,}489.0$
16-25	5284	1179	22.3%	$p < 0.001$
26-35	5785	1461	25.3%	
36-45	4947	1685	34.1%	
46-55	5004	2108	42.1%	
56-65	3083	1337	43.4%	
66-75	1299	461	35.5%	
76-85	882	166	18.8%	
+86	328	36	11.0%	

For peer review only

For peer review only - http://bmjopen.bmj.com/site/about/guidelines.xhtml

Missing	37	0	0.0%	
Index of Multiple Deprivation**				
0 <= x <= 20 - (Most deprived)	11019	2205	20.0%	$\chi^2(1)$ = 18,720.0
20 < x <= 30	10265	3066	29.9%	$p < 0.001$
30 < x <= 93 - (Least deprived)	10963	3461	31.6%	
Missing	1252	370	29.6%	
Most Recent Primary Diagnosis via ICD10 code				
F0x - Organic, including symptomatic, mental disorders	631	125	19.8%	$\chi^2(12)$ =11,085.74
F1x - Mental and behavioural disorders due to psychoactive substance use	2271	696	30.6%	$p < 0.001$
F2x - Schizophrenia, schizotypal and delusional disorders	5230	3910	74.8%	
F3x - Mood [affective] disorders	2798	1232	44.0%	
F4x - Neurotic, stress-related and somatoform disorders	2424	596	24.6%	
F5x - Behavioural syndromes associated with physiological disturbances and physical factors	543	144	26.5%	
F6x - Disorders of adult personality and behaviour	1165	611	52.4%	

For peer review only

F7x - Mental retardation	484	283	58.5%	
F8x - Disorders of psychological developmental	1418	413	29.1%	
F9x - Behavioural and emotional disorders with onset usually occurring in childhood and adolescence	2187	521	23.8%	
Zx - Unspecified	280	86	30.7%	
Any other letter x	200	38	19.0%	
Not recorded	13868	447	3.2%	
Any inpatient care in the preceding 12 months				
No	33042	8746	26.5%	$\chi^2(1) = 602.52$
Yes	457	356	77.9%	$p < 0.001$
Number of face to face community contacts in the preceding 12 months**				
0	16895	1589	9.4%	$\chi^2(1)$ = 20,984.0
1-7	7434	2166	29.1%	$p < 0.001$
8-14	3347	1629	48.7%	

For peer review only

15-21	2277	1350	59.3%	
22-28	1431	916	64.0%	
29-42	1292	866	67.0%	
+43	823	586	71.2%	
Year of first SLAM referral**				
0 before 2007	6110	4494	73.6%	$\chi^2(1)$ = 12,894.0
1 2007 - 2008	1932	1058	54.8%	$p < 0.001$
2 2009 - 2010	1781	1121	62.9%	
3 2011 - 2012	2028	989	48.8%	
4 2013 - 2014	2469	333	13.5%	
5 2015 - 2016	3789	331	8.7%	
6 2017 - 2018	6138	399	6.5%	
7 2019 - 2020	8995	178	2.0%	
Missing	257	199	77.4%	

For peer review only - http://bmjopen.bmj.com/site/about/guidelines.xhtml

+ 'Missing' category has been removed when running chi-square tests

**For ordinal variables, chi-squared text for linear trends ($\chi 2(1)$) was conducted

For peer review only

For peer review only - http://bmjopen.bmj.com/site/about/guidelines.xhtml

Table 4. Unadjusted and adjusted odds ratios for each characteristic as an independent factor in the presence of quoted text

	Unadjusted Odds Ratio (95% CI)		Adjusted Odds Ratio (95% CI)* (complete cases, n = 24, 381)	
Gender (reference = Female)				
Male	1.21	(1.15,1.27)	1.02	(0.94,1.10)
Ethnicity (reference = White European)				
Black	2.20	(2.08,2.33)	1.28	(1.18,1.39)
Asian	1.19	(1.06,1.34)	1.03	(0.87,1.21)
Other	0.86	(0.77,0.96)	0.89	(0.77,1.04)
Age group	1.24	(1.23,1.26)	0.89	(0.87,0.92)

For peer review only - http://bmjopen.bmj.com/site/about/guidelines.xhtml

Index of Multiple Deprivation	1.34	(1.30,1.38)	1.01	(0.97,1.06)
Most Recent Primary Diagnosis via ICD10 code (reference = F2x)				
F0x - Organic, including symptomatic, mental disorders	0.08	(0.07,0.10)	0.42	(0.32,0.54)
F1x - Mental and behavioural disorders due to psychoactive substance use	0.15	(0.13,0.17)	0.33	(0.29,0.38)
F3x - Mood [affective] disorders	0.27	(0.24,0.29)	0.54	(0.47,0.61)
F4x - Neurotic, stress-related and somatoform disorders	0.11	(0.10,0.12)	0.38	(0.33,0.44)
F5x - Behavioural syndromes associated with physiological disturbances and physical factors	0.12	(0.10,0.15)	0.51	(0.40,0.65)
F6x - Disorders of adult personality and behaviour	0.37	(0.33,0.42)	0.63	(0.54,0.75)
F7x - Mental retardation	0.48	(0.39,0.58)	0.57	(0.46,0.72)
F8x - Disorders of psychological developmental	0.14	(0.12,0.16)	0.44	(0.37,0.53)
F9x - Behavioural and emotional disorders with onset usually occurring in childhood and adolescence	0.11	(0.09,0.12)	0.40	(0.34,0.48)
Zx - Unspecified	0.15	(0.12,0.19)	0.48	(0.34,0.67)
Any other letter x	0.08	(0.06,0.11)	0.27	(0.18,0.42)

For peer review only - http://bmjopen.bmj.com/site/about/guidelines.xhtml

For peer review only

Not recorded	0.01	(0.01,0.01)	0.16	(0.14,0.19)
Any inpatient care in the preceding 12 months (reference = No)				
Yes	9.79	(7.84,12.23)	3.77	(2.78,5.11)
Number of face to face community contacts in the preceding 12 months	1.90	(1.86,1.93)	1.30	(1.27,1.33)
Year of first SLAM referral	0.52	(0.51,0.52)	0.58	(0.57,0.59)

*Adjusted for gender, ethnicity, age group, index of multiple deprivation, most recent primary diagnosis, any inpatient care in the preceding 12 months,

number of face to face community contacts in the preceding 12 months and year of first SLAM referral.

For peer review only - http://bmjopen.bmj.com/site/about/guidelines.xhtml

Table 5a. The twenty most frequent words in each k-means cluster generated using the CRIS general discharge summary word-embeddings and their subjective category label

Group assigned by k-means cluster	0	1	2	3
Subjective label	miscellaneous	mental illness	sentiment	verbs
Twenty most frequent words	friend	no	ok	voice
	correspondence	yes	fine	they
	right	high	good	nothing
	boring	breakdown	normal	everything
	lost	stress	alright	people
	lazy	manic	okay	thing
	on	problem	happy	help
	spirit	depression	better	something

For peer review only - http://bmjopen.bmj.com/site/about/guidelines.xhtml

	bastard	paranoia	well	them
	hello	mental	safe	control
	fit	crisis	stable	hate
	free	episode	nice	it
	bitch	illness	bad	dead
	perfect	psychotic	depressed	attack
	blip	failure	mad	work
	prn	anxiety	low	kill
	crap	pressure	paranoid	fight
	goodbye	pain	stuck	know
	home	anger	stressed	worry
	flat	heavy	stupid	rule

For peer review only - http://bmjopen.bmj.com/site/about/guidelines.xhtml

Table 5b. The twenty most frequent words in each k-means cluster generated using the CRIS early psychosis intervention services word-embeddings and their subjective category label

Group assigned by k-means	0	1*	2	3	4	5	6	7	8
Subjective label	mental illness	verbs	verbs	negative sentiment	people/relationships	mixed sentiment	aggression/violence	healthcare	negative connotation
Twenty most frequent words	no	rule	help	normal	friend	ok	lost	yes	terrible
	breakdown	break	control	high	people	fine	on	correspondence	boring
	stress	turn	work	depressed	flat	good	rude	mental	horrible
	problem	go	kill	low	mum	voice	dead	waiting	rubbish
	depression	cut	know	paranoid	boyfriend	alright	gay	should	bastard
	paranoia	sorted	worry	angry	baby	bad	attack	crisis	hello
	event	run	stop	hyper	girlfriend	okay	fight	carer	fat
	episode	gone	escape	unwell	family	happy	forced	assessment	free
	illness	jump	leave	confused	dad	better	contaminated	need	shit

For peer review only - http://bmjopen.bmj.com/site/about/guidelines.xhtml

	psychotic	dropped	treat	manic	mother	mad	bullied	duty	bitch
	anxiety	drop	lie	strange	someone	well	accident	counselling	useless
	pressure	fall	hurt	stable	old	stuck	missing	therapy	perfect
	pain	character	feel	odd	partner	stressed	failed	nurse	awful
	anger	fell	relax	anxious	uncle	stupid	attacked	plan	blip
	heavy		push	calm	sister	they	sectioned	care	prn
	issue		see	positive	man	down	hit	we	crap
	emotional		die	feeling	relationship	sad	lying	urgent	goodbye
	psychosis		change	suicidal	child	nothing	talking	freedom	naughty
	panic		do	thought	husband	bored	fighting	respite	flashback
	seizure		poison	calmer	girl	safe	controlling	trust	rough

* Only 14 words were present in total in this group

For peer review only - http://bmjopen.bmj.com/site/about/guidelines.xhtml

40/ 42

For peer review only

41/ 42

For peer review only

AUTHOR STATEMENT

Contributors: RS and LJ conceived the study design with advice from SV. LJ wrote the paper and analysed the data. RS provided supervisory guidance. All authors provided critical input for the paper and approved the submission.

Funding: LJ, SV and RS are part-funded by the National Institute for Health Research (NIHR) Biomedical Research Centre at the South London and Maudsley NHS Foundation Trust and King's College London. RS is additionally funded by i) a Medical Research Council (MRC) Mental Health Data Pathfinder Award to King's College London; ii) an NIHR Senior Investigator Award; iii) the National Institute for Health Research (NIHR) Applied Research Collaboration South London (NIHR ARC South London) at King's College Hospital NHS Foundation Trust. The views expressed are those of the authors and not necessarily those of the NIHR or the Department of Health and Social Care.

Competing interests: RS has received research support in the last 5 years from Janssen, GSK and Takeda.

Ethics approval: Oxford C Research Ethics Committee (reference 18/SC/0372).

Data sharing statement: Data must remain within the SLaM firewall and any requests to access the data can be addressed to cris.administrator@kcl.ac.uk.

For peer review only

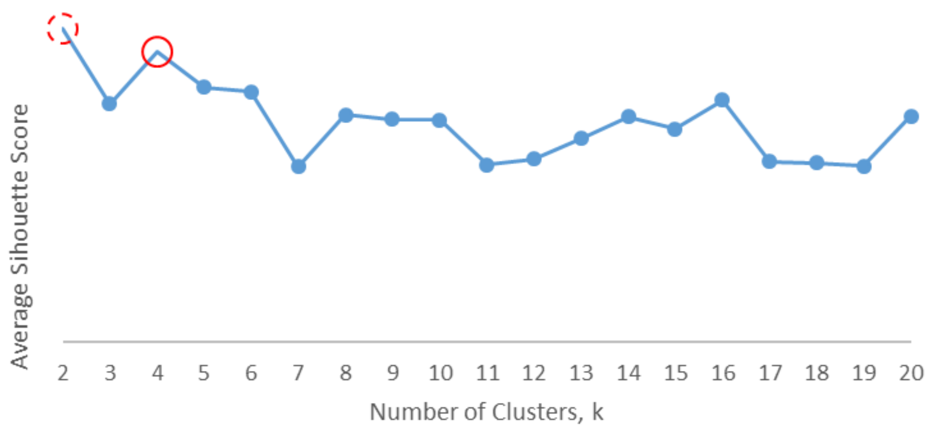

Figure 1. Average sihouette score for clusters generated from CRIS general discharge summaries word-embeddings

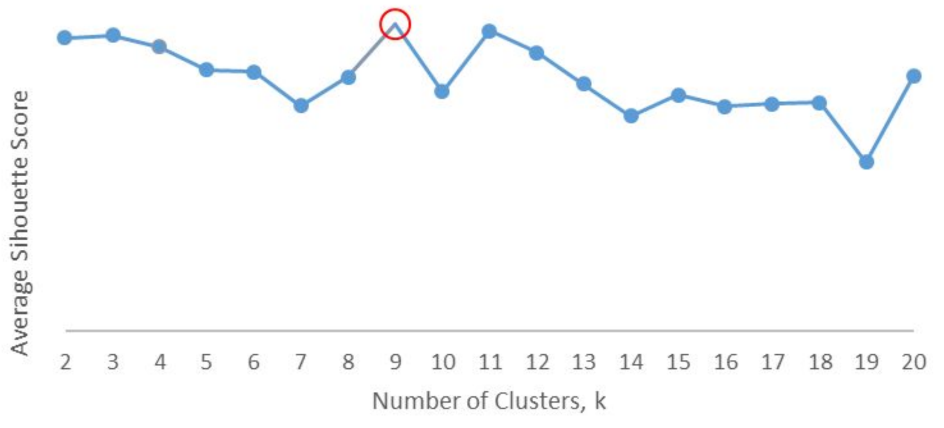

Figure 2. Average sihouette score for clusters generated from CRIS early psychosis intervention services word-embeddings

The RECORD statement – checklist of items, extended from the STROBE statement, that should be reported in observational studies using routinely collected health data.

	Item No.	STROBE items	Location in manuscript where items are reported	RECORD items	Location in manuscript where items are reported
**Title and abstract**					
	1	(a) Indicate the study's design with a commonly used term in the title or the abstract (b) Provide in the abstract an informative and balanced summary of what was done and what was found		RECORD 1.1: The type of data used should be specified in the title or abstract. When possible, the name of the databases used should be included.<br><br>RECORD 1.2: If applicable, the geographic region and timeframe within which the study took place should be reported in the title or abstract.<br><br>RECORD 1.3: If linkage between databases was conducted for the study, this should be clearly stated in the title or abstract.	Title and abstract<br><br>Abstract<br><br>N/A
**Introduction**					
Background rationale	2	Explain the scientific background and rationale for the investigation being reported			Introduction paragraphs 1 - 3
Objectives	3	State specific objectives, including any prespecified hypotheses			Introduction Paragraph 3
**Methods**					
Study Design	4	Present key elements of study design early in the paper			Methods: study sample, The distribution of individuals with quoted

					text, Variables, The content of quoted text
Setting	5	Describe the setting, locations, and relevant dates, including periods of recruitment, exposure, follow-up, and data collection			Methods: study sample
Participants	6	*(a) Cohort study* - Give the eligibility criteria, and the sources and methods of selection of participants. Describe methods of follow-up<br>*Case-control study* - Give the eligibility criteria, and the sources and methods of case ascertainment and control selection. Give the rationale for the choice of cases and controls<br>*Cross-sectional study* - Give the eligibility criteria, and the sources and methods of selection of participants<br><br>*(b) Cohort study* - For matched studies, give matching criteria and number of exposed and unexposed<br>*Case-control study* - For matched studies, give matching criteria and the number of controls per case		RECORD 6.1: The methods of study population selection (such as codes or algorithms used to identify subjects) should be listed in detail. If this is not possible, an explanation should be provided.<br><br>RECORD 6.2: Any validation studies of the codes or algorithms used to select the population should be referenced. If validation was conducted for this study and not published elsewhere, detailed methods and results should be provided.<br><br>RECORD 6.3: If the study involved linkage of databases, consider use of a flow diagram or other graphical display to demonstrate the data linkage process, including the number of individuals with linked data at each stage.	Cross-sectional study<br>Methods: study sample section<br>The distribution of individuals with quoted text, Variables, The content of quoted text<br>N/A<br><br>N/A
Variables	7	Clearly define all outcomes, exposures, predictors, potential confounders, and effect modifiers. Give diagnostic criteria, if applicable.		RECORD 7.1: A complete list of codes and algorithms used to classify exposures, outcomes, confounders, and effect modifiers should be provided. If these cannot be reported, an explanation should be provided.	Methods: study sample section<br>The distribution of individuals with quoted text, Variables, The

For peer review only - http://bmjopen.bmj.com/site/about/guidelines.xhtml

					content of quoted text
Data sources/ measurement	8	For each variable of interest, give sources of data and details of methods of assessment (measurement). Describe comparability of assessment methods if there is more than one group			
Bias	9	Describe any efforts to address potential sources of bias			N/A
Study size	10	Explain how the study size was arrived at			Methods: study sample & Statistical analysis
Quantitative variables	11	Explain how quantitative variables were handled in the analyses. If applicable, describe which groupings were chosen, and why			Methods: Variables
Statistical methods	12	(a) Describe all statistical methods, including those used to control for confounding<br>(b) Describe any methods used to examine subgroups and interactions<br>(c) Explain how missing data were addressed<br>(d) *Cohort study* - If applicable, explain how loss to follow-up was addressed<br>*Case-control study* - If applicable, explain how matching of cases and controls was addressed			a) Methods, statistical analysis<br>b) N/A<br>c) Methods: statistical analysis<br>d) N/A<br>e) N/A

For peer review only

		Cross-sectional study - If applicable, describe analytical methods taking account of sampling strategy<br>(e) Describe any sensitivity analyses			
Data access and cleaning methods		..		RECORD 12.1: Authors should describe the extent to which the investigators had access to the database population used to create the study population.<br><br>RECORD 12.2: Authors should provide information on the data cleaning methods used in the study.	Methods: study sample<br><br>Methods: statistical analysis
Linkage		..		RECORD 12.3: State whether the study included person-level, institutional-level, or other data linkage across two or more databases. The methods of linkage and methods of linkage quality evaluation should be provided.	N/A
**Results**					
Participants	13	(a) Report the numbers of individuals at each stage of the study (*e.g.*, numbers potentially eligible, examined for eligibility, confirmed eligible, included in the study, completing follow-up, and analysed)<br>(b) Give reasons for non-participation at each stage.<br>(c) Consider use of a flow diagram		RECORD 13.1: Describe in detail the selection of the persons included in the study (*i.e.,* study population selection) including filtering based on data quality, data availability and linkage. The selection of included persons can be described in the text and/or by means of the study flow diagram.	Methods: study sample section
Descriptive data	14	(a) Give characteristics of study participants (*e.g.*, demographic, clinical, social) and information			Results: Table 2. & 3.

		on exposures and potential confounders<br>(b) Indicate the number of participants with missing data for each variable of interest<br>(c) *Cohort study* - summarise follow-up time (*e.g.*, average and total amount)			
Outcome data	15	*Cohort study* - Report numbers of outcome events or summary measures over time<br>*Case-control study* - Report numbers in each exposure category, or summary measures of exposure<br>*Cross-sectional study* - Report numbers of outcome events or summary measures			Table 3.
Main results	16	(a) Give unadjusted estimates and, if applicable, confounder-adjusted estimates and their precision (e.g., 95% confidence interval). Make clear which confounders were adjusted for and why they were included<br>(b) Report category boundaries when continuous variables were categorized<br>(c) If relevant, consider translating estimates of relative risk into absolute risk for a meaningful time period			Table 4.
Other analyses	17	Report other analyses done—e.g., analyses of subgroups and interactions, and sensitivity analyses			Table 5a.<br>Table 5b.
**Discussion**					

For peer review only

Key results	18	Summarise key results with reference to study objectives			Discussion, paragraph 1, 6
Limitations	19	Discuss limitations of the study, taking into account sources of potential bias or imprecision. Discuss both direction and magnitude of any potential bias		RECORD 19.1: Discuss the implications of using data that were not created or collected to answer the specific research question(s). Include discussion of misclassification bias, unmeasured confounding, missing data, and changing eligibility over time, as they pertain to the study being reported.	Discussion, paragraph 5
Interpretation	20	Give a cautious overall interpretation of results considering objectives, limitations, multiplicity of analyses, results from similar studies, and other relevant evidence			Discussion, paragraph 2 -3
Generalisability	21	Discuss the generalisability (external validity) of the study results			Discussion, paragraph 2 -3
**Other Information**					
Funding	22	Give the source of funding and the role of the funders for the present study and, if applicable, for the original study on which the present article is based			Funding statement
Accessibility of protocol, raw data, and programming code		..		RECORD 22.1: Authors should provide information on how to access any supplemental information such as the study protocol, raw data, or programming code.	Data sharing statement

*Reference: Benchimol EI, Smeeth L, Guttmann A, Harron K, Moher D, Petersen I, Sørensen HT, von Elm E, Langan SM, the RECORD Working Committee. The REporting of studies Conducted using Observational Routinely-collected health Data (RECORD) Statement. *PLoS Medicine* 2015; in press.

*Checklist is protected under Creative Commons Attribution (CC BY) license.

For peer review only

BMJ Open

Quoted Text in the Mental Health Care Electronic Record: An Analysis of the Distribution and Content of Single-Word Quotations

Journal:	*BMJ Open*
Manuscript ID	bmjopen-2021-049249.R1
Article Type:	Original research
Date Submitted by the Author:	03-Aug-2021
Complete List of Authors:	Jayasinghe, Lasantha; King's College London Institute of Psychiatry Psychology and Neuroscience, Department of Psychological Medicine<br>Velupillai, Sumithra; King's College London Institute of Psychiatry Psychology and Neuroscience, Department of Psychological Medicine<br>Stewart, Robert; King's College London Institute of Psychiatry Psychology and Neuroscience, Department of Psychological Medicine; South London and Maudsley NHS Foundation Trust
**Primary Subject Heading**:	Health informatics
Secondary Subject Heading:	Mental health
Keywords:	MENTAL HEALTH, PSYCHIATRY, Health informatics < BIOTECHNOLOGY & BIOINFORMATICS

SCHOLARONE™
Manuscripts

BMJ

I, the Submitting Author has the right to grant and does grant on behalf of all authors of the Work (as defined in the below author licence), an exclusive licence and/or a non-exclusive licence for contributions from authors who are: i) UK Crown employees; ii) where BMJ has agreed a CC-BY licence shall apply, and/or iii) in accordance with the terms applicable for US Federal Government officers or employees acting as part of their official duties; on a worldwide, perpetual, irrevocable, royalty-free basis to BMJ Publishing Group Ltd ("BMJ") its licensees and where the relevant Journal is co-owned by BMJ to the co-owners of the Journal, to publish the Work in this journal and any other BMJ products and to exploit all rights, as set out in our licence.

The Submitting Author accepts and understands that any supply made under these terms is made by BMJ to the Submitting Author unless you are acting as an employee on behalf of your employer or a postgraduate student of an affiliated institution which is paying any applicable article publishing charge ("APC") for Open Access articles. Where the Submitting Author wishes to make the Work available on an Open Access basis (and intends to pay the relevant APC), the terms of reuse of such Open Access shall be governed by a Creative Commons licence – details of these licences and which Creative Commons licence will apply to this Work are set out in our licence referred to above.

Other than as permitted in any relevant BMJ Author's Self Archiving Policies, I confirm this Work has not been accepted for publication elsewhere, is not being considered for publication elsewhere and does not duplicate material already published. I confirm all authors consent to publication of this Work and authorise the granting of this licence.

For peer review only

Quoted Text in the Mental Health Care Electronic Record: An Analysis of the Distribution and Content of Single-Word Quotations

Lasantha Jayasinghe[1], Sumithra Velupillai[1], Robert Stewart[*,1,2]
[1] *Institute of Psychiatry, Psychology and Neuroscience, King's College London, London, UK,*
[2] *South London and Maudsley NHS Foundation Trust, London, UK*

Correspondence to: Lasantha Jayasinghe. lasantha.jayasinghe@kcl.ac.uk

Other Author emails:
Sumithra Velupillai: Sumithra.Velupillai @kcl.ac.uk
Robert Stewart: robert.stewart@kcl.ac.uk

Keywords: electronic health records, mental health, natural language processing, unsupervised learning, k-means clustering

Word count: 3,887

For peer review only

For peer review only - http://bmjopen.bmj.com/site/about/guidelines.xhtml

ABSTRACT

Objective:

To investigate the distribution and content of quoted text within the electronic health records (EHR) using a previously developed NLP tool to generate a database of quotations.

Design:

Chi-squared and logistic regression were used to assess the profile of patients receiving mental healthcare for whom quotations exist. K-means clustering using pre-trained word embeddings developed on general discharge summaries and psychosis specific mental health records were used to group one-word quotations into semantically similar groups and labelled by human subjective judgement.

Setting:

Electronic health records from a large mental healthcare provider serving a geographic catchment area of 1.3 million residents in South London.

Participants:

For analysis of distribution, 33,499 individuals receiving mental healthcare on the 30th June 2019 in South London and Maudsley (SLaM). For analysis of content, 1,587 unique lemmatized words, appearing a minimum of 20 times on the database of quotations created on the 16th January 2020.

Results:

The strongest individual indicator of quoted text is inpatient care in the preceding 12 months (OR 9.79, 95% CI 7.84 - 12.23). Next highest indicator is ethnicity with those with a black background more likely to have quoted text in comparison to white background (OR 2.20, 95% CI 2.08 - 2.33). Both are attenuated slightly in the adjusted model. Early psychosis intervention word-embeddings subjectively produced categories pertaining to: mental illness, verbs, negative sentiment, people/relationships, mixed sentiment, aggression/violence and negative connotation

Conclusions:

For peer review only - http://bmjopen.bmj.com/site/about/guidelines.xhtml

The findings that inpatients and those from a black ethnic background more commonly have quoted text raise important questions around where clinical attention is focused and whether this may point to any systematic bias. Our study also shows that word embeddings trained on early psychosis intervention records are useful in categorizing even small subsets of the clinical records represented by one-word quotations.

For peer review only

For peer review only - http://bmjopen.bmj.com/site/about/guidelines.xhtml

ARTICLE SUMMARY

Strengths and limitations of this study

- A large sample size (n= 33,499) for logistic regression of all patients receiving mental healthcare on the 30th June 2019 was used in this study and comparisons made between characteristics of groups with/without a quotation.
- Pre-trained word-embeddings used to categorise one-word quotations based on a large electronic mental health record corpus of around 23 million words each.
- 27% (9,118) of the data for those with quotations on the 30th June 2019 contained variables with missing values and therefore were not included in the adjusted logistic regression analysis
- Investigator subjective judgement is used to determine the category label of clusters and consequently the optimum number of clusters.

For peer review only

For peer review only - http://bmjopen.bmj.com/site/about/guidelines.xhtml

INTRODUCTION

Mental health electronic health records (EHRs) routinely capture a wealth of unstructured information detailing a patient's clinical journey including symptoms, observed behaviours, contextual factors, assessments, interventions and outcomes within the free-text fields of case notes and correspondence (1). The majority of studies using this information have focused on these clinical constructs (2). However the EHR is also a narrative account written from the perspective of healthcare professionals (3). Within this account, clinicians often add exact quotes from patient testimony and other parties, for example as evidence for their diagnosis or other decisions (4,5). Quoted text in the EHR has the potential to give insight into the types of information recorded by clinicians and into the patient voice, albeit as secondary information filtered through the lens of the clinician, reflecting both the focus of the encounter and the individual clinician's reporting style(6,7). This is of particular interest in two respects.

First, due to the lack of standardisation of clinical reporting (8) it is unknown to what extent there is coherence in clinician testimony as represented by quoted text and how this relates to outcomes for patients. In this respect, many previous studies, rather than examining quoted speech directly, have looked at instances of 'referencing', where the source of text is assigned to a third party using the 'he/she says' construct. In one study there was a greater relative frequency in third-person pronoun use in a group of veterans who had died from suicide, in contrast to a comparison service user group (9), and another study found an increase in referencing when doctors communicated negative news to patients (10). On the other hand, a previous study by our group found that the frequency of quoted text in the EHR did not change in the time period nearer a suicide attempt, indicating that clinicians did not change the frequency of directly reporting patient testimony even when patients symptoms may have worsened (7).

For peer review only - http://bmjopen.bmj.com/site/about/guidelines.xhtml

Second, incorporating the patient voice in the EHR has become a growing area of interest (11,12) with data from new studies indicating that inclusion of electronic patient-reported outcomes (ePROs) is associated with improved levels of patient care in areas such as cancer treatment(13). Given this context, the examination of the content of quoted text pre-existing in the EHR at least represents the beginnings of wider inclusion of the patient voice while ePROs are under development. However, little or no research has been carried out to date on such text.

As a precursor to understanding the content of quoted text, it is important to understand the patient populations from whom quotations are taken. It is currently unknown, for example, which patients are more likely to have quoted text in their EHR, or if there are variations between different demographic or diagnostic groups. Therefore, building on our previous work to ascertain quoted text at scale in the full EHR, the first objective of this study was to ascertain a fine-grain understanding of the distribution of quoted text within patients receiving secondary mental healthcare by undertaking analysis of frequencies by key demographic and clinical characteristics.. Second, we sought to investigate the content of the quoted text itself. Again, to the best of our knowledge, the content of quoted text in EHRs is largely unknown, limiting conclusions that can be drawn. For example, it is unknown whether quotes predominantly relate to psychopathological terms that clinicians have been trained to note down, or whether they cover other indications of patient experience outside medical terminology.

Due to the large volumes of the data, we opted to approach the problem using natural language processing (NLP) methods and to apply k-means clustering to extracted text, an unsupervised method, with the aim of classifying the quotations. NLP has increasingly been used to extract clinically relevant information such as symptoms and medication from EHRs (14), and as part of the work to investigate quoted text frequencies in relation to self-harm, we had already developed an

For peer review only - http://bmjopen.bmj.com/site/about/guidelines.xhtml

algorithm to identify and extract these text strings from a large mental healthcare EHR (7). One way of representing words is via word embeddings, where semantically similar words have similar numerical vector representations (15). These vectors are generated by applying machine learning models over specific text corpora. Previous studies on the same mental healthcare platform as our data have generated word embeddings to identify terminology around general symptoms of mental illness (16) and more specifically psychosis (17). Focusing on one-word quotations in the first instance, as the most common form, we sought to address the deficiency of information on content and to investigate the extent to which these pre-trained embeddings might be used to classify the quotations identified from a mental healthcare EHR.

For peer review only

For peer review only - http://bmjopen.bmj.com/site/about/guidelines.xhtml

METHODS

Study sample

The South London and Maudsley NHS Foundation Trust (SLaM) provides comprehensive, near-monopoly mental healthcare services to a geographic catchment of around 1.3m residents in four boroughs of south London, as well as some national specialist services. The mental health records used in this study were assembled using SLaM's Clinical Record Interactive Search (CRIS) platform, which currently accesses mental healthcare records for over 500,000 patients, rendering them de-identified and accessible for research use (18). CRIS, as a data resource for secondary analysis, has IRB approval from the Oxford Research Ethics Committee C (reference 18/SC/0372).

The distribution of individuals with quoted text

An overview of the methodology is given in Figure 1.The first objective of the study was to describe the distribution of those with quoted text at the database creation date, 16th January 2020, on which there were 365,555 total active patients in SLaM from which quotations would potentially arise. The date of the first instance of quoted text for a patient was used as the index date to determine the variable values. Additionally, for comparison purposes we extracted the same variables for all active SLaM patients on a particular index date, 30th June 2019 and compared the people with or without quoted text to see if there were any differences.

Variables

All variables were derived from structured text fields at index date. The basic demographic variables were gender, ethnicity and age. For analytical purposes, we summarized ethnicity into five groups, as follows: White European (British, Irish, Any other white background), Black (African, White and Black African, Caribbean, White and Black Caribbean, Any other black background), Asian (Bangladeshi, Indian, Pakistani, Chinese, Any other Asian background, White and Asian), Other (Any

For peer review only - http://bmjopen.bmj.com/site/about/guidelines.xhtml

other mixed background, Any other ethnic group) and Missing. Other variables included were: the Index of Multiple Deprivation Score, most recent primary diagnosis, whether any inpatient care was received in the preceding 12 months with a binary Yes/No outcome, the number of community face to face contacts in the preceding 12 months and the year of first patient referral to SLaM.

The Index of Multiple Deprivation (IMD) Score (19) represents the socioeconomic status of neighborhoods in the UK by combining various economic, social and housing indicators. It is based on the 2011 national census data and calculated from the patient's most recent address at index date and the distribution of national percentile scores for given neighbourhoods has been most commonly categorised by tertiles in previous CRIS analyses (20–22); thus the same categories were applied here for consistency ($0 <= x <= 20$, $20 < x <= 30$, $30 < x <= 93$), with lower scored groups indicating greater deprivation. The most recent primary diagnosis was determined from the ICD10 code assigned to the patient at index date. In this analysis, the groups were represented by the first letter and/or digit of the ICD10 code, resulting in the following categories: F0x, F1x, F2x, F3x, F4x, F5x, F6x, F7x, F8x, F9x, Zx, Any other letter x and Not recorded.

The content of quoted text

The second objective of our study was to investigate the content of quoted text. Previous work by our team involved the development of a natural language processing (NLP) tool to identify text occurring within quotation marks in the EHR using regular expression matching on a sample of patients hospitalised for a suicide attempt (7). The details of the application have been previously described (7), but in summary this algorithm yielded a precision of 0.92, recall of 0.93 against a manually annotated gold standard. As one-word quotations were the largest proportion by word count, these 199,384 instances (27% of the total) were chosen as the focus for this particular investigation.

For peer review only - http://bmjopen.bmj.com/site/about/guidelines.xhtml

Statistical analysis

Data and statistical analysis were performed using standard Python (version 3.6.8) libraries. To analyse the distribution of individuals with quoted text, first the characteristics for all individuals with quoted text at the database creation date of 16th January 2020 were calculated. Where an individual had more than one instance of quoted text, the date of the first quotation was used for variable extraction.

Second, in the sample of patients receiving mental healthcare on 30th June 2019, chi-squared tests were used to test the associations between each individual variable and the presence of quoted text. Chi-squared tests were calculated with missing values excluded and for ordinal variables (age group, index of multiple deprivation, number of face to face community contacts, year of first SLaM referral) as a linear trend with one degree of freedom. Then logistic regression was used to analyse whether individual variables increased the likelihood of the presence of quotations and whether this was attenuated when all variables were incorporated in a single model. The logistic regression analyses were conducted using complete cases only.

To analyse the content of quoted text, first the one-word quotations (199,384) were extracted from the quotations database, lower-cased and lemmatized (converted to its base form) using the NLTK package, WordNetLemmatizer (23) . This resulted in 25,873 unique lemmatized words. Of these, the most frequent quotations, classified as those appearing at least 20 times, were compiled into a list for further analysis, giving 1,587 words. These were then mapped into vector format via the word embeddings and used in the clustering algorithm. We used the pre-trained word embeddings generated by Viani and colleagues (17) trained specifically on CRIS records pertaining to 1) general discharge summaries (23.6m words) reflecting all mental health disorder records (not restricted to

For peer review only - http://bmjopen.bmj.com/site/about/guidelines.xhtml

psychosis) and 2) early psychosis intervention services (23.3 m words) across all mental health services. These were trained using a gensim (24) implementation of Word2Vec with the Continuous Bag of Words (CBOW) model. We felt this was a suitable approach as these embeddings have been specifically trained on CRIS records, so are more likely to reflect the semantic similarity of words in the context specific to our mental health platform. For example, words such as 'hyper' are used more loosely in general terminology, but have specific meanings in the clinical context.

The idea behind clustering methodology is to use an unsupervised algorithm to group together semantically similar words as represented by their vector forms. K-means was chosen as it has high accuracy and speed with large datasets and provides good data segmentation (25). The implementation of k-means used was Python's *sklearn* library, which by default runs the algorithm ten times with different centroid seeds to minimize the impact of clusters forming around local minima. Several methods are available to select the optimum number of clusters. We initially sought to use the 'elbow method' to determine the number of clusters by plotting the inertia for 1 to 20 clusters(26). However, as no obvious 'elbow' was apparent, we opted for silhouette analysis which examines the separation distance between each k-means cluster (26,27). The higher the average silhouette coefficients, the further the clusters are apart, with a maximum value of 1. Under this method the higher the average silhouette score for each cluster, the better the representation of the data. Therefore, in statistical terms, the optimum number of clusters is represented by the highest average silhouette score over a range of possible values. This was determined by plotting the average silhouette scores for 1 to 20 clusters and examining the highest values. Our objective was to classify the content of one-word quotations in a way that added meaning to the group; it was therefore necessary to use subjective judgement to assess whether the optimum number of clusters provided distinct meaningful groups. To assess the usefulness of the k-means clustering algorithm in assigning semantically similar words to similar groups, the investigator examined the 20 most

For peer review only

For peer review only - http://bmjopen.bmj.com/site/about/guidelines.xhtml

frequent words in each cluster and, using subjective judgement (the lead investigator's initial decision followed by consensus achieved in the research group), assigned a descriptive label for each cluster. If at least one cluster could be given a homogeneous label, then the process was complete, and this number of clusters was deemed optimum. Otherwise, the number of clusters with the next highest average silhouette score was assessed in a similar way, and so on until it was possible for the investigator to assess at least one of the clusters as a homogenous group.

Patient involvement

We did not directly incorporate patient and public involvement (PPI) into this particular study, but the SLaM BRC Case Register used in the analysis was developed with extensive PPI and is overseen by a committee that includes service-user representatives.

For peer review only

For peer review only - http://bmjopen.bmj.com/site/about/guidelines.xhtml

RESULTS

The previously developed tool (7) to identify quoted text from a sample of CRIS records was run over the whole CRIS database, which contained 365,555 records on the 16th January 2020. After removal of blank quotes and those representing ‘html’ tag data, 728,505 quotations were identified from CRIS, relating to 53,476 individuals. The quotations were further categorised in terms of word count. The mean number of words in a quotation was 9, median 3, standard deviation 25 and range 1-309, indicating a wide distribution in the size of quotations with a positive skew. Details for the volumes of quotations by word length are described in Table 1.

Characteristics of patients with at least one instance of quoted text are displayed in Table 2. Characteristics of the total number of patients in receipt of SLaM care on the 30th June 2019 index date are further shown in Table 3. alongside the subset of patients with quoted text. Quoted text was more common in male patients, in those from black compared to white ethnic groups and those living in more deprived neighbourhoods. In terms of clinical variables, quoted text was more common in those with schizophrenia, schizotypal and delusional disorders (F2x), those receiving inpatient care, and those with higher numbers of community face to face contacts in the preceding 12 months.

The unadjusted and adjusted logistic regression results are presented in Table 4. The presence of any inpatient care in the preceding 12 months is the strongest individual indicator of quoted text, with those receiving care nearly ten times more likely to have quoted text than those without. In terms of ethnicity, black individuals are approximately twice as likely as white Europeans to have instances of quoted text, although this is attenuated by the presence of other variables in the adjusted model. In comparison to the reference category, F2x (Schizophrenia, schizotypal and delusional disorders), other primary diagnoses are very unlikely to produce instances of quoted text. Additionally, gender,

For peer review only - http://bmjopen.bmj.com/site/about/guidelines.xhtml

age group and Index of Multiple Deprivation have very little effect on the presence of quoted text, in the adjusted model.

Cluster Analyses

The optimum number of clusters suggested by silhouette analysis (see Figure 2) for the discharge summary word embeddings was two. This yielded two clusters, which appeared to distinguish between a group referring to sentiment (negative and positive) and a miscellaneous group. As the investigator observed that both groups appeared to contain mixed rather than distinct categories, the next highest silhouette score was examined and this yielded four clusters, which are displayed in Table 5. This shows that group 0 is miscellaneous with no obvious descriptive category label, whilst the other groups appear to contain words related to mental illness, sentiment and verbs. The optimum number of clusters using the early intervention word embeddings was nine (see Figure 3), as shown in Table 6. These groups appeared to contain more clearly differentiable categories, relating to mental illness, verbs (2 groups), negative sentiment, people/relationships, mixed sentiment, aggression/violence and negative connotation.

For peer review only

For peer review only - http://bmjopen.bmj.com/site/about/guidelines.xhtml

DISCUSSION

To the best of our knowledge, this is the first study to describe the distribution of quoted text and the content of one-word length quotations from a clinical record database, the size of which adds strength to our findings. In a sample of SLaM patients at census date 30th June 2019, those with any inpatient care in the preceding 12 months are most likely to have quoted text in the clinical record, even after adjusting for other variables. Ethnicity was the next most pertinent factor, with quoted text appearing more commonly for those with a black ethnic background, but this was attenuated slightly in the adjusted model. Individuals with schizophrenia, schizotypal and delusional disorders (F2x) were much more likely to have quoted text than those with any other primary diagnosis, although primary diagnosis in general had little effect in the adjusted model. This study also found that one-word quotations were clustered into more distinctive categories using the early intervention word embeddings in comparison to those generated from discharge summaries. This resulted in nine groups which could be subjectively labelled as follows: mental illness, verbs (2 groups), negative sentiment, people/relationships, mixed sentiment, aggression/violence and negative connotation.

As described, the relevant contexts for this study are the increasing volume of data now being routinely collected in EHR resources, and the growing awareness of the potential utility for such data to support research and improved clinical practice and/or service configuration, alongside the fact that EHRs reflect primarily a clinician's perspective and authorship. Although quotations in the text remain filtered by that perspective, they do at least provide the beginnings of a 'patient voice' in the EHR while systems for direct patient input to the health record are developed. Given the lack of information on quoted text, even basic information such as the frequency of its recording and the characteristics of patients and/or services/contexts in which it is recorded, we sought to compile some preliminary data on distribution and on the content of single-word quotations as the most

For peer review only - http://bmjopen.bmj.com/site/about/guidelines.xhtml

common type observed. This drew on earlier published work to ascertain such quotations automatically and at scale across the EHR through natural language processing (7).

Our findings of any inpatient care in the preceding 12 months being the strongest indicator of quoted text may be due to those hospitalised receiving more frequent clinical observation than outpatients, leading to a greater volume of clinical notes from which quotations may arise. Further, there may be a greater focus on quoting text as evidence for decision making and medical defence practice (4,5), given that inpatients are more likely to be suffering from the most severe mental health conditions. On a similar basis, a greater frequency of quoted text from those of black ethnicity may be explained by higher levels of psychosis being present in this group in comparison to white ethnic backgrounds (28–30) and consequently these individuals may receive a greater clinical focus.

The finding that the early psychosis intervention word-embeddings produce more distinct categories in the data makes sense in the context of around 44% of all quotations in CRIS arising from patients with a recent primary diagnosis of F1x - Mental and behavioural disorders due to psychoactive substance use, F2x - Schizophrenia, schizotypal and delusional disorders, or F3x - Mood [affective] disorders, as all these have psychosis as a possible symptom. Therefore, word-embeddings trained on records most similar to those from which the quotations are derived are likely to produce the best results in the clustering process. It is interesting to note that aside from categories related to mental illness and sentiment, this study has uncovered other more unexpected areas where clinicians may quote their patients, in terms of aggression/violence, people/relationships and verbs, indicating an emphasis on the circumstances of a patient’s experience rather than purely symptomatology.

For peer review only

For peer review only - http://bmjopen.bmj.com/site/about/guidelines.xhtml

Strengths and limitations

This study has several strengths. First it examines a novel area by focussing on the distribution and content of quotations within the EHR rather than the full record, giving insight into information clinicians may quote beyond clinical terminology. Furthermore, the large sample sizes for analysis of both distribution and content add significance to the findings. Additionally, the word-embeddings used to represent the one-word quotations have been trained on millions of words which are highly relevant since they have been derived specifically from mental health records on the same platform.

The findings of our study need to be taken with several limitations in mind. One limitation of our study is that categories applied may be heterogeneous, for example the ethnic groups selected. Another limitation is that 27% of the sample data were incomplete cases and therefore were not included in the adjusted logistic regression analysis. Another consideration is that data for logistic regression were examined at one point in time, so unknown confounders may be present in the data, such as previous service use for a different mental health disorder. In terms of investigating the content of quoted text, one key limitation is that the labelling of groups found by clustering is subjective and based on the assessment of the researcher. Another key limitation is that what is found in the text is dependent on what the clinician notes down; this will be subject to training and individual preferences and biases. Additionally, attribution of the speaker is not determined by the algorithm although the majority of quotations were from patients (7). Further, as we chose to investigate one-word quotations as a first step, the meaning derived from words in terms of clustering is limited without context. Therefore, further studies should examine longer strings of quotations to gauge a better understanding of content. Additionally, further studies could use contextual word-vector representations. Under this methodology, words are assigned vector representations based on the surrounding contextual words, to give a better idea of how a specific word is used in a particular context.

For peer review only

For peer review only - http://bmjopen.bmj.com/site/about/guidelines.xhtml

Conclusions

Despite limitations, this is an important study as the first of its kind to investigate the profile of patients and the areas of patient experience that are highlighted in quoted speech within the clinical record. The successful creation of a database across all CRIS to identify quoted speech is a first step in making this data available for future studies. The findings that inpatients and those from a black ethnic background more commonly have quoted text raise important questions around where clinical attention is focused and whether this may point to any systematic bias. Our study also shows that word embeddings trained on early psychosis intervention records are useful in categorizing small subsets of the clinical records represented by one-word quotations.

For peer review only

For peer review only - http://bmjopen.bmj.com/site/about/guidelines.xhtml

REFERENCES

1. Perera G, Broadbent M, Callard F, Chang CK, Downs J, Dutta R, et al. Cohort profile of the South London and Maudsley NHS Foundation Trust Biomedical Research Centre (SLaM BRC) Case Register: Current status and recent enhancement of an Electronic Mental Health Record-derived data resource. BMJ Open. 2016 Mar 1;6(3):e008721.
2. Velupillai S, Hadlaczky G, Baca-Garcia E, Gorrell GM, Werbeloff N, Nguyen D, et al. Risk Assessment Tools and Data-Driven Approaches for Predicting and Preventing Suicidal Behavior. Front psychiatry [Internet]. 2019 [cited 2019 Jun 19];10:36. Available from: http://www.ncbi.nlm.nih.gov/pubmed/30814958
3. Aaslestad P. The patient as text: the role of the narrator in psychiatric notes, 1890-1990. CRC Press; 2016.
4. GOV.UK. Assessing and managing risk in mental health services - GOV.UK [Internet]. 2009 [cited 2019 Oct 9]. Available from: https://www.gov.uk/government/publications/assessing-and-managing-risk-in-mental-health-services
5. Roth, Lorraine S. Writing progress notes: 10 dos and don’ts [Internet]. MDedge Psychiatry. 2005 [cited 2019 Oct 9]. p. 4(2):63-66. Available from: https://www.mdedge.com/psychiatry/article/59861/writing-progress-notes-10-dos-and-donts
6. Walsh J, Cominos N, Jureidini J. 13. Patient voice: including the patient in mental health handovers. Eff Commun Clin Handover. 2016 Mar 19;
7. Jayasinghe L, Bittar A, Dutta R, Stewart R. Clinician-recalled quoted speech in electronic health records and risk of suicide attempt: A case-crossover study. BMJ Open. 2020 Apr 22;10(4):e036186.
8. Chung AE, Basch EM. Incorporating the patient’s voice into electronic health records through patient-reported outcomes as the “review of systems.” J Am Med Informatics Assoc

For peer review only - http://bmjopen.bmj.com/site/about/guidelines.xhtml

[Internet]. 2015 Jul 1 [cited 2021 Jul 28];22(4):914–6. Available from: https://academic.oup.com/jamia/article/22/4/914/1745633

9. Leonard Westgate C, Shiner B, Thompson P, Watts B V. Evaluation of Veterans' Suicide Risk With the Use of Linguistic Detection Methods. Psychiatr Serv [Internet]. 2015 Oct 15 [cited 2019 Jun 5];66(10):1051–6. Available from: http://psychiatryonline.org/doi/10.1176/appi.ps.201400283

10. Van DeMieroop D. The quotative 'he/she says' in interpreted doctor–patient interaction. Interpreting [Internet]. 2012 Jan 1 [cited 2019 Jun 19];14(1):92–117. Available from: http://www.jbe-platform.com/content/journals/10.1075/intp.14.1.05mie

11. Nordan L, Blanchfield L, Niazi S, Sattar J, Coakes CE, Uitti R, et al. Implementing electronic patient-reported outcomes measurements: challenges and success factors. BMJ Qual Saf [Internet]. 2018 Oct 1 [cited 2021 Jul 29];27(10):852–6. Available from: https://qualitysafety.bmj.com/content/27/10/852

12. Harle CA, Listhaus A, Covarrubias CM, Schmidt SO, Mackey S, Carek PJ, et al. Overcoming barriers to implementing patient-reported outcomes in an electronic health record: a case report. J Am Med Informatics Assoc [Internet]. 2016 Jan 1 [cited 2021 Jul 29];23(1):74–9. Available from: https://academic.oup.com/jamia/article/23/1/74/2379954

13. LeBlanc TW, Abernethy AP. Patient-reported outcomes in cancer care — hearing the patient voice at greater volume. Nat Rev Clin Oncol 2017 1412 [Internet]. 2017 Oct 4 [cited 2021 Jul 28];14(12):763–72. Available from: https://www.nature.com/articles/nrclinonc.2017.153

14. Wang Y, Wang L, Rastegar-Mojarad M, Moon S, Shen F, Afzal N, et al. Clinical information extraction applications: A literature review. Vol. 77, Journal of Biomedical Informatics. Academic Press Inc.; 2018. p. 34–49.

15. Mikolov T, Chen K, Corrado G, Dean J. Efficient estimation of word representations in vector space. In: 1st International Conference on Learning Representations, ICLR 2013 - Workshop

For peer review only

Track Proceedings. International Conference on Learning Representations, ICLR; 2013.

16. Stewart R, Jackson R, Patel R, Velupillai S, Gkotsis G, Hoyle D. Knowledge discovery for Deep Phenotyping serious mental illness from Electronic Mental Health records [version 2; referees: 2 approved]. F1000Research. 2018;7.

17. Viani N, Patel R, Stewart R, Velupillai S. Generating positive psychosis symptom keywords from electronic health records. In: Lecture Notes in Computer Science (including subseries Lecture Notes in Artificial Intelligence and Lecture Notes in Bioinformatics). Springer Verlag; 2019. p. 298–303.

18. Perera G, Broadbent M, Callard F, Chang C-K, Downs J, Dutta R, et al. Cohort profile of the South London and Maudsley NHS Foundation Trust Biomedical Research Centre (SLaM BRC) Case Register: current status and recent enhancement of an Electronic Mental Health Record-derived data resource. BMJ Open [Internet]. 2016 Mar 1 [cited 2019 Jun 19];6(3):e008721. Available from: http://www.ncbi.nlm.nih.gov/pubmed/26932138

19. Gov. English indices of deprivation 2019 - GOV.UK [Internet]. [cited 2020 Nov 16]. Available from: https://www.gov.uk/government/statistics/english-indices-of-deprivation-2019

20. Fok ML-Y, Stewart R, Hayes RD, Moran P. Predictors of Natural and Unnatural Mortality among Patients with Personality Disorder: Evidence from a Large UK Case Register. PLoS One [Internet]. 2014 Jul 7 [cited 2021 Jul 28];9(7):e100979. Available from: https://journals.plos.org/plosone/article?id=10.1371/journal.pone.0100979

21. Roberts E, Wessely S, Chalder T, Chang CK, Hotopf M. Mortality of people with chronic fatigue syndrome: a retrospective cohort study in England and Wales from the South London and Maudsley NHS Foundation Trust Biomedical Research Centre (SLaM BRC) Clinical Record Interactive Search (CRIS) Register. Lancet. 2016 Apr 16;387(10028):1638–43.

22. McQuarrie EP, Mackinnon B, Bell S, Fleming S, McNeice V, Stewart G, et al. Multiple socioeconomic deprivation and impact on survival in patients with primary

For peer review only - http://bmjopen.bmj.com/site/about/guidelines.xhtml

glomerulonephritis. Clin Kidney J [Internet]. 2017 Feb 1 [cited 2021 Jul 28];10(1):49. Available from: /pmc/articles/PMC5469556/

23. NLTK. nltk.stem.wordnet — NLTK 3.5 documentation [Internet]. [cited 2021 Jan 6]. Available from: https://www.nltk.org/_modules/nltk/stem/wordnet.html

24. Gensim. gensim · PyPI [Internet]. [cited 2021 Jan 6]. Available from: https://pypi.org/project/gensim/

25. Rodriguez MZ, Comin CH, Casanova D, Bruno OM, Amancio DR, Costa L da F, et al. Clustering algorithms: A comparative approach. PLoS One [Internet]. 2019 Jan 1 [cited 2020 Aug 3];14(1):e0210236. Available from: https://doi.org/10.1371/journal.pone.0210236

26. (PDF) Review on Determining of Cluster in K-means Clustering [Internet]. [cited 2021 Jul 26]. Available from: https://www.researchgate.net/publication/313554124_Review_on_Determining_of_Cluster_in_K-means_Clustering

27. Determining The Optimal Number Of Clusters: 3 Must Know Methods - Datanovia [Internet]. [cited 2021 Jun 21]. Available from: https://www.datanovia.com/en/lessons/determining-the-optimal-number-of-clusters-3-must-know-methods/#average-silhouette-method

28. Veling W. Ethnic minority position and risk for psychotic disorders. Curr Opin Psychiatry [Internet]. 2013 Mar [cited 2020 Dec 2];26(2):166–71. Available from: http://journals.lww.com/00001504-201303000-00007

29. Coid JW, Kirkbride JB, Barker D, Cowden F, Stamps R, Yang M, et al. Raised incidence rates of all psychoses among migrant groups: Findings from the east london first episode psychosis study. Arch Gen Psychiatry [Internet]. 2008 Nov 3 [cited 2020 Dec 2];65(11):1250–8. Available from: https://jamanetwork.com/journals/jamapsychiatry/fullarticle/482871

30. Kirkbride JB, Hameed Y, Ioannidis K, Ankireddypalli G, Crane CM, Nasir M, et al. Ethnic Minority Status, Age-at-Immigration and Psychosis Risk in Rural Environments: Evidence From

For peer review only - http://bmjopen.bmj.com/site/about/guidelines.xhtml

the SEPEA Study. Schizophr Bull [Internet]. 2017 Oct 21 [cited 2020 Dec 2];43(6):1251–61. Available from: http://academic.oup.com/schizophreniabulletin/article/43/6/1251/3829518

For peer review only

For peer review only - http://bmjopen.bmj.com/site/about/guidelines.xhtml

FIGURE LEGENDS

Figure 1. Overview of methodology workflow

Figure 2. Average silhouette score for clusters generated from CRIS general discharge summaries word-embeddings

Figure 3. Average silhouette score for clusters generated from CRIS early psychosis intervention services word-embeddings

For peer review only

For peer review only - http://bmjopen.bmj.com/site/about/guidelines.xhtml

TABLES AND FIGURES

Table 1. The word length of quotations in the database

Word length	N	% Total
0	1694	0.23
1	199,384	27.37
2	148,394	20.37
3	86,476	11.87
4	58,993	8.1
5	42,482	5.83
6	30,791	4.23
7	22,616	3.1
8	18,066	2.48
9	13,581	1.86
10 - 14	37,566	5.17
15 - 19	15,936	2.19
20 - 24	8,205	1.13
25 - 29	5,347	0.73
30 - 34	3,854	0.53
35 - 39	3,036	0.41
40 - 44	2,487	0.35
45 - 49	2,233	0.3
50 - 54	1,951	0.26
55 - 74	6,106	0.84
75 - 99	5,129	0.7
100 - 149	6,713	0.69
150 - 199	4,028	0.68
200 +	3,437	0.68

For peer review only

For peer review only - http://bmjopen.bmj.com/site/about/guidelines.xhtml

Table 2. Characteristics of patients receiving mental healthcare who have at least one quotation on the index date (16th January 2020)

	All patients on the Quotations database*	
	N	% Total
**Gender**		
Female	26420	49.4%
Male	27035	50.6%
Unknown	21	0.0%
**Ethnicity**		
White European	31435	58.8%
Black	14155	26.5%
Asian	2592	4.8%
Other	3456	6.5%
Unknown	1838	3.4%
**Age group**		
1-15	2463	4.6%
16-25	10363	19.4%
26-35	7417	13.9%
36-45	8484	15.9%
46-55	9198	17.2%
56-65	5942	11.1%
66-75	2976	5.6%
76-85	2751	5.1%
+86	3872	7.2%
Unknown	10	0.0%
**Index of Multiple Deprivation**		
0 <= x <= 20 - (Most deprived)	15386	28.8%
20 < x <= 30	17026	31.8%
30 < x <= 93 - (Least deprived)	19323	36.1%
Unknown	1741	3.3%
**Most Recent Primary Diagnosis via ICD10 code**		
F0x - Organic, including symptomatic, mental disorders	4853	9.1%
F1x - Mental and behavioural disorders due to psychoactive substance use	6065	11.3%
F2x - Schizophrenia, schizotypal and delusional disorders	8773	16.4%

For peer review only - http://bmjopen.bmj.com/site/about/guidelines.xhtml

F3x - Mood [affective] disorders	8773	16.4%
F4x - Neurotic, stress-related and somatoform disorders	6248	11.7%
F5x - Behavioural syndromes associated with physiological disturbances and physical factors	1958	3.7%
F6x - Disorders of adult personality and behaviour	2548	4.8%
F7x - Mental retardation	1145	2.1%
F8x - Disorders of psychological developmental	2037	3.8%
F9x - Behavioural and emotional disorders with onset usually occurring in childhood and adolescence	3578	6.7%
Zx - Unspecified	768	1.4%
Any other letter x	376	0.7%
Not recorded	6354	11.9%
**Any inpatient care in the preceding 12 months**		
No	52986	99.1%
Yes	490	0.9%
**Number of face to face community contacts in the preceding 12 months**		
0	40518	75.8%
1-7	5666	10.6%
8-14	2521	4.7%
15-21	1848	3.5%
22-28	1162	2.2%
29-42	1067	2.0%
+43	694	1.3%
**Year of first SLAM referral**		
1918 - 2014	50339	94.1%
2015 - 2020	2580	4.8%
Unknown	557	1.0%

*Where a patient had more than one quotation data has been derived from the date of their earliest record

For peer review only

Table 3. Characteristics of patients receiving mental healthcare on the index date (30th June 2019, n= 33,499) alongside a comparison of the subset of patients with at least one instance of quoted text on the 30th June 2019

	All Patients Active on SLAM at 30th June 2019	Patients with quotations Active on SLAM at 30th June 2019		
	N	N	% All SLAM Active Patients by Row Total	Chi-Square tests of independence+
**Gender**				
Female	15992	4039	25.30%	χ2(1) = 57.81
Male	17459	5056	29.00%	p < 0.001
Missing	48	7	14.60%	
**Ethnicity**				
White European	14560	4263	29.30%	χ2(3) = 822.63
Black	7752	3694	47.70%	p < 0.001
Asian	1440	476	33.10%	
Other	1864	488	26.20%	
Missing	7883	181	2.30%	
**Age group****				
1-15	6850	669	9.80%	χ2(1) = 28,489.0
16-25	5284	1179	22.30%	p < 0.001
26-35	5785	1461	25.30%	
36-45	4947	1685	34.10%	
46-55	5004	2108	42.10%	
56-65	3083	1337	43.40%	
66-75	1299	461	35.50%	
76-85	882	166	18.80%	
86	328	36	11.00%	
Missing	37	0	0.00%	
**Index of Multiple Deprivation****				
0 <= x <= 20 - (Most deprived)	11019	2205	20.00%	χ2(1) = 18,720.0
20 < x <= 30	10265	3066	29.90%	p < 0.001
30 < x <= 93 - (Least deprived)	10963	3461	31.60%	
Missing	1252	370	29.60%	
**Most Recent Primary Diagnosis via ICD10 code**				
F0x - Organic, including symptomatic, mental disorders	631	125	19.80%	χ2(12) =11,085.74
F1x - Mental and behavioural disorders due to psychoactive substance use	2271	696	30.60%	p < 0.001
F2x - Schizophrenia, schizotypal and delusional disorders	5230	3910	74.80%	
F3x - Mood [affective] disorders	2798	1232	44.00%	

For peer review only - http://bmjopen.bmj.com/site/about/guidelines.xhtml

F4x - Neurotic, stress-related and somatoform disorders	2424	596	24.60%	
F5x - Behavioural syndromes associated with physiological disturbances and physical factors	543	144	26.50%	
F6x - Disorders of adult personality and behaviour	1165	611	52.40%	
F7x - Mental retardation	484	283	58.50%	
F8x - Disorders of psychological developmental	1418	413	29.10%	
F9x - Behavioural and emotional disorders with onset usually occurring in childhood and adolescence	2187	521	23.80%	
Zx - Unspecified	280	86	30.70%	
Any other letter x	200	38	19.00%	
Not recorded	13868	447	3.20%	
**Any inpatient care in the preceding 12 months**				
No	33042	8746	26.50%	χ2(1) = 602.52
Yes	457	356	77.90%	p < 0.001
**Number of face to face community contacts in the preceding 12 months****				
0	16895	1589	9.40%	χ2(1) = 20,984.0
1-7	7434	2166	29.10%	p < 0.001
8-14	3347	1629	48.70%	
15-21	2277	1350	59.30%	
22-28	1431	916	64.00%	
29-42	1292	866	67.00%	
43	823	586	71.20%	
**Year of first SLAM referral****				
before 2007	6110	4494	73.60%	χ2(1) = 12,894.0
2007 - 2008	1932	1058	54.80%	p < 0.001
2009 - 2010	1781	1121	62.90%	
2011 - 2012	2028	989	48.80%	
2013 - 2014	2469	333	13.50%	
2015 - 2016	3789	331	8.70%	
2017 - 2018	6138	399	6.50%	
2019 - 2020	8995	178	2.00%	
Missing	257	199	77.40%	

+ 'Missing' category has been removed when running chi-square tests
**For ordinal variables, chi-squared text for linear trends (χ2(1)) was conducted

For peer review only

Table 4. Unadjusted and adjusted odds ratios for each characteristic as an independent factor in the presence of quoted text

	Unadjusted Odds Ratio (95% CI)		Adjusted Odds Ratio (95% CI)* (complete cases, n = 24, 381)	
**Gender** (reference = Female)				
Male	1.21	(1.15,1.27)	1.02	(0.94,1.10)
**Ethnicity** (reference = White European)				
Black	2.2	(2.08,2.33)	1.28	(1.18,1.39)
Asian	1.19	(1.06,1.34)	1.03	(0.87,1.21)
Other	0.86	(0.77,0.96)	0.89	(0.77,1.04)
**Age group**	1.24	(1.23,1.26)	0.89	(0.87,0.92)
**Index of Multiple Deprivation**	1.34	(1.30,1.38)	1.01	(0.97,1.06)
**Most Recent Primary Diagnosis via ICD10 code (reference = F2x)**				
F0x - Organic, including symptomatic, mental disorders	0.08	(0.07,0.10)	0.42	(0.32,0.54)
F1x - Mental and behavioural disorders due to psychoactive substance use	0.15	(0.13,0.17)	0.33	(0.29,0.38)
F3x - Mood [affective] disorders	0.27	(0.24,0.29)	0.54	(0.47,0.61)
F4x - Neurotic, stress-related and somatoform disorders	0.11	(0.10,0.12)	0.38	(0.33,0.44)
F5x - Behavioural syndromes associated with physiological disturbances and physical factors	0.12	(0.10,0.15)	0.51	(0.40,0.65)
F6x - Disorders of adult personality and behaviour	0.37	(0.33,0.42)	0.63	(0.54,0.75)
F7x - Mental retardation	0.48	(0.39,0.58)	0.57	(0.46,0.72)
F8x - Disorders of psychological developmental	0.14	(0.12,0.16)	0.44	(0.37,0.53)
F9x - Behavioural and emotional disorders with onset usually occurring in childhood and adolescence	0.11	(0.09,0.12)	0.4	(0.34,0.48)
Zx - Unspecified	0.15	(0.12,0.19)	0.48	(0.34,0.67)
Any other letter x	0.08	(0.06,0.11)	0.27	(0.18,0.42)
Not recorded	0.01	(0.01,0.01)	0.16	(0.14,0.19)
**Any inpatient care in the preceding 12 months (reference = No)**				
Yes	9.79	(7.84,12.23)	3.77	(2.78,5.11)

For peer review only

**Number of face to face community contacts in the preceding 12 months**	1.9	(1.86,1.93)	1.3	(1.27,1.33)
**Year of first SLAM referral**	0.52	(0.51,0.52)	0.58	(0.57,0.59)

*Adjusted for gender, ethnicity, age group, index of multiple deprivation, most recent primary diagnosis, any inpatient care in the preceding 12 months, number of face to face community contacts in the preceding 12 months and year of first SLAM referral.

For peer review only

For peer review only - http://bmjopen.bmj.com/site/about/guidelines.xhtml

Table 5. The twenty most frequent words in each k-means cluster generated using the CRIS general discharge summary word-embeddings and their subjective category label

Group assigned by k-means cluster	0	1	2	3
Subjective label	miscellaneous	mental illness	sentiment	verbs
Twenty most frequent words	friend	no	ok	voice
	correspondence	yes	fine	they
	right	high	good	nothing
	boring	breakdown	normal	everything
	lost	stress	alright	people
	lazy	manic	okay	thing
	on	problem	happy	help
	spirit	depression	better	something
	bastard	paranoia	well	them
	hello	mental	safe	control
	fit	crisis	stable	hate
	free	episode	nice	it
	bitch	illness	bad	dead
	perfect	psychotic	depressed	attack
	blip	failure	mad	work
	prn	anxiety	low	kill
	crap	pressure	paranoid	fight
	goodbye	pain	stuck	know
	home	anger	stressed	worry
	flat	heavy	stupid	rule

For peer review only - http://bmjopen.bmj.com/site/about/guidelines.xhtml

Table 6. The twenty most frequent words in each k-means cluster generated using the CRIS early psychosis intervention services word-embeddings and their subjective category label

Group assigned by k-means	0	1*	2	3	4	5	6	7	8
Subjective label	mental illness	verbs	verbs	negative sentiment	people/ relationships	mixed sentiment	aggression/ violence	healthcare	negative connotation
Twenty most frequent words	no	rule	help	normal	friend	ok	lost	yes	terrible
	breakdown	break	control	high	people	fine	on	correspondence	boring
	stress	turn	work	depressed	flat	good	rude	mental	horrible
	problem	go	kill	low	mum	voice	dead	waiting	rubbish
	depression	cut	know	paranoid	boyfriend	alright	gay	should	bastard
	paranoia	sorted	worry	angry	baby	bad	attack	crisis	hello
	event	run	stop	hyper	girlfriend	okay	fight	carer	fat
	episode	gone	escape	unwell	family	happy	forced	assessment	free
	illness	jump	leave	confused	dad	better	contaminated	need	shit
	psychotic	dropped	treat	manic	mother	mad	bullied	duty	bitch
	anxiety	drop	lie	strange	someone	well	accident	counselling	useless
	pressure	fall	hurt	stable	old	stuck	missing	therapy	perfect
	pain	character	feel	odd	partner	stressed	failed	nurse	awful
	anger	fell	relax	anxious	uncle	stupid	attacked	plan	blip
	heavy		push	calm	sister	they	sectioned	care	prn
	issue		see	positive	man	down	hit	we	crap
	emotional		die	feeling	relationship	sad	lying	urgent	goodbye
	psychosis		change	suicidal	child	nothing	talking	freedom	naughty
	panic		do	thought	husband	bored	fighting	respite	flashback
	seizure		poison	calmer	girl	safe	controlling	trust	rough

* Only 14 words were present in total in this group

34/ 35

For peer review only

AUTHOR STATEMENT

Contributors: RS and LJ conceived the study design with advice from SV. LJ wrote the paper and analysed the data. RS provided supervisory guidance. All authors provided critical input for the paper and approved the submission.

Funding: LJ, SV and RS are part-funded by the National Institute for Health Research (NIHR) Biomedical Research Centre at the South London and Maudsley NHS Foundation Trust and King's College London. RS is additionally funded by i) a Medical Research Council (MRC) Mental Health Data Pathfinder Award to King's College London; ii) an NIHR Senior Investigator Award; iii) the National Institute for Health Research (NIHR) Applied Research Collaboration South London (NIHR ARC South London) at King's College Hospital NHS Foundation Trust. The views expressed are those of the authors and not necessarily those of the NIHR or the Department of Health and Social Care.

Competing interests: RS has received research support in the last 5 years from Janssen, GSK and Takeda.

Ethics approval: Oxford C Research Ethics Committee (reference 18/SC/0372).

Data sharing statement: Data must remain within the SLaM firewall and any requests to access the data can be addressed to cris.administrator@kcl.ac.uk.

For peer review only

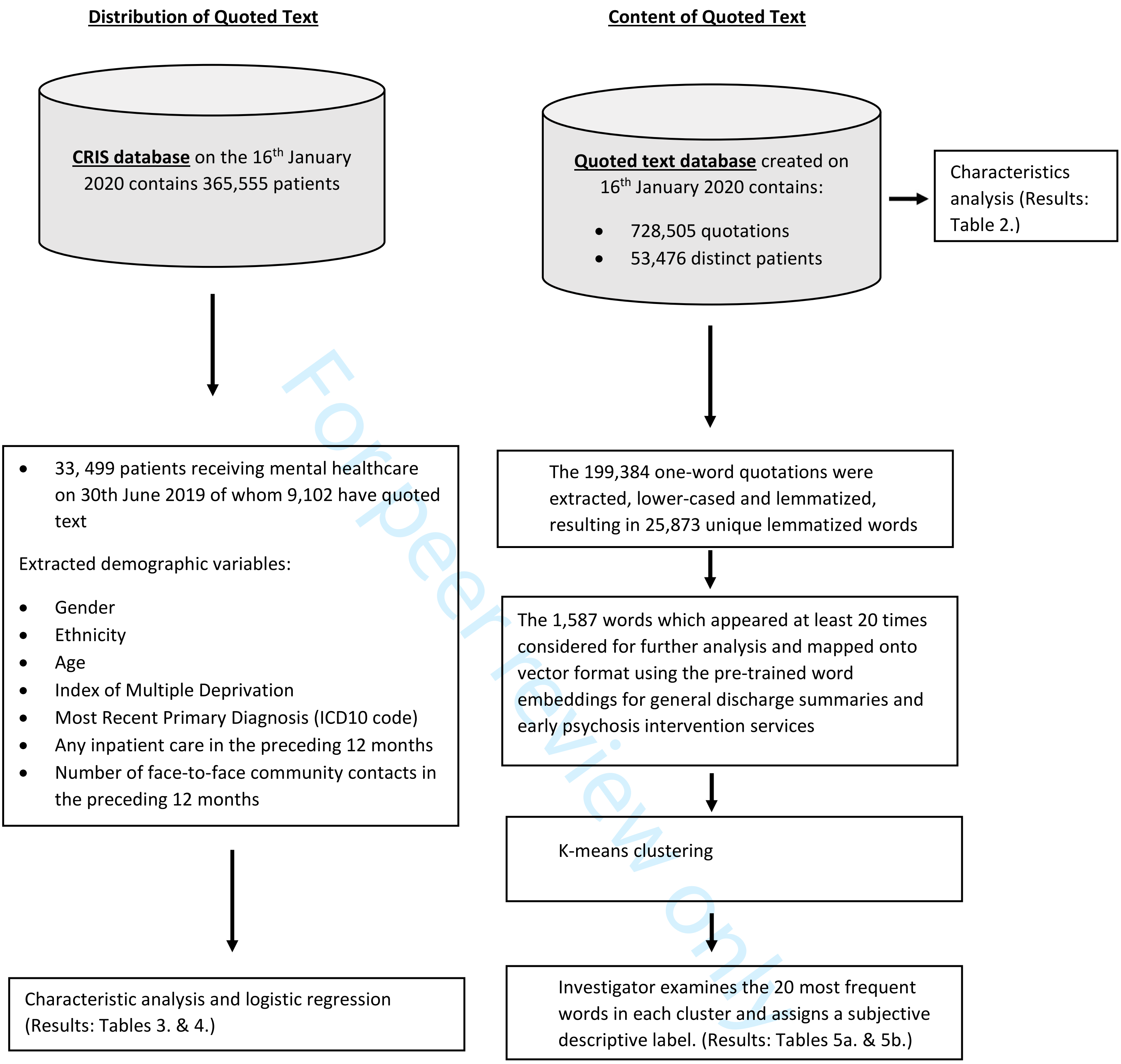

For peer review only

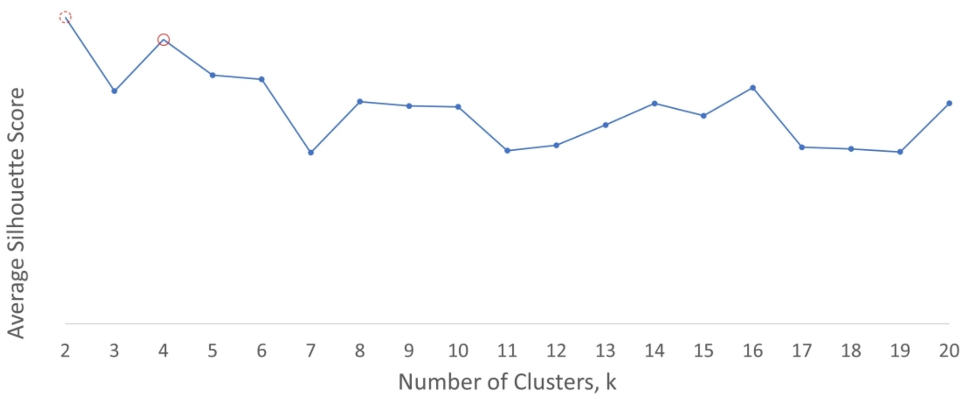

Figure 2. Average silhouette score for clusters generated from CRIS general discharge summaries word-embeddings

338x190mm (300 x 300 DPI)

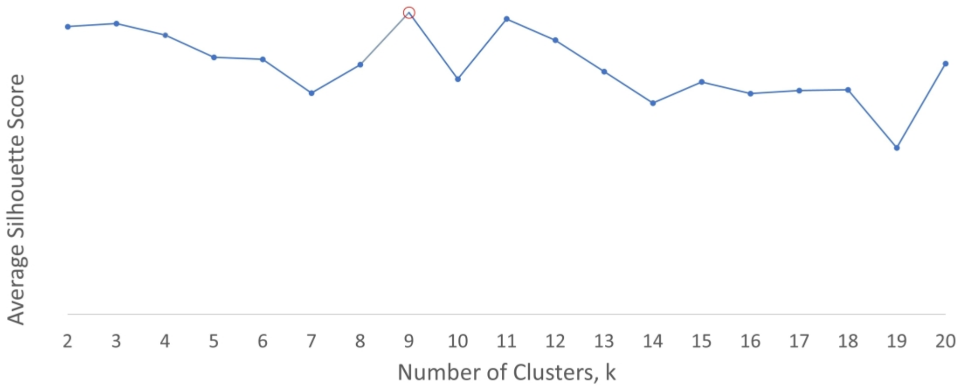

Figure 3. Average silhouette score for clusters generated from CRIS early psychosis intervention services word-embeddings

338x190mm (300 x 300 DPI)

The RECORD statement – checklist of items, extended from the STROBE statement, that should be reported in observational studies using routinely collected health data.

	Item No.	STROBE items	Location in manuscript where items are reported	RECORD items	Location in manuscript where items are reported
**Title and abstract**					
	1	(a) Indicate the study's design with a commonly used term in the title or the abstract (b) Provide in the abstract an informative and balanced summary of what was done and what was found		RECORD 1.1: The type of data used should be specified in the title or abstract. When possible, the name of the databases used should be included.<br><br>RECORD 1.2: If applicable, the geographic region and timeframe within which the study took place should be reported in the title or abstract.<br><br>RECORD 1.3: If linkage between databases was conducted for the study, this should be clearly stated in the title or abstract.	Title and abstract<br><br>Abstract<br><br>N/A
**Introduction**					
Background rationale	2	Explain the scientific background and rationale for the investigation being reported			Introduction paragraphs 1 - 3
Objectives	3	State specific objectives, including any prespecified hypotheses			Introduction Paragraph 3
**Methods**					
Study Design	4	Present key elements of study design early in the paper			Methods: study sample, The distribution of individuals with quoted

For peer review only

					text, Variables, The content of quoted text
Setting	5	Describe the setting, locations, and relevant dates, including periods of recruitment, exposure, follow-up, and data collection			Methods: study sample
Participants	6	*(a) Cohort study* - Give the eligibility criteria, and the sources and methods of selection of participants. Describe methods of follow-up<br>*Case-control study* - Give the eligibility criteria, and the sources and methods of case ascertainment and control selection. Give the rationale for the choice of cases and controls<br>*Cross-sectional study* - Give the eligibility criteria, and the sources and methods of selection of participants<br><br>*(b) Cohort study* - For matched studies, give matching criteria and number of exposed and unexposed<br>*Case-control study* - For matched studies, give matching criteria and the number of controls per case		RECORD 6.1: The methods of study population selection (such as codes or algorithms used to identify subjects) should be listed in detail. If this is not possible, an explanation should be provided.<br><br>RECORD 6.2: Any validation studies of the codes or algorithms used to select the population should be referenced. If validation was conducted for this study and not published elsewhere, detailed methods and results should be provided.<br><br>RECORD 6.3: If the study involved linkage of databases, consider use of a flow diagram or other graphical display to demonstrate the data linkage process, including the number of individuals with linked data at each stage.	Cross-sectional study<br>Methods: study sample section<br>The distribution of individuals with quoted text, Variables, The content of quoted text<br>N/A<br><br>N/A
Variables	7	Clearly define all outcomes, exposures, predictors, potential confounders, and effect modifiers. Give diagnostic criteria, if applicable.		RECORD 7.1: A complete list of codes and algorithms used to classify exposures, outcomes, confounders, and effect modifiers should be provided. If these cannot be reported, an explanation should be provided.	Methods: study sample section<br>The distribution of individuals with quoted text, Variables, The

For peer review only

					content of quoted text
Data sources/ measurement	8	For each variable of interest, give sources of data and details of methods of assessment (measurement).<br>Describe comparability of assessment methods if there is more than one group			
Bias	9	Describe any efforts to address potential sources of bias			N/A
Study size	10	Explain how the study size was arrived at			Methods: study sample & Statistical analysis
Quantitative variables	11	Explain how quantitative variables were handled in the analyses. If applicable, describe which groupings were chosen, and why			Methods: Variables
Statistical methods	12	(a) Describe all statistical methods, including those used to control for confounding<br>(b) Describe any methods used to examine subgroups and interactions<br>(c) Explain how missing data were addressed<br>(d) *Cohort study* - If applicable, explain how loss to follow-up was addressed<br>*Case-control study* - If applicable, explain how matching of cases and controls was addressed			a) Methods, statistical analysis<br>b) N/A<br>c) Methods: statistical analysis<br>d) N/A<br>e) N/A

For peer review only

		Cross-sectional study - If applicable, describe analytical methods taking account of sampling strategy<br>(e) Describe any sensitivity analyses			
Data access and cleaning methods		..		RECORD 12.1: Authors should describe the extent to which the investigators had access to the database population used to create the study population.<br><br>RECORD 12.2: Authors should provide information on the data cleaning methods used in the study.	Methods: study sample<br><br>Methods: statistical analysis
Linkage		..		RECORD 12.3: State whether the study included person-level, institutional-level, or other data linkage across two or more databases. The methods of linkage and methods of linkage quality evaluation should be provided.	N/A
**Results**					
Participants	13	(a) Report the numbers of individuals at each stage of the study (*e.g.*, numbers potentially eligible, examined for eligibility, confirmed eligible, included in the study, completing follow-up, and analysed)<br>(b) Give reasons for non-participation at each stage.<br>(c) Consider use of a flow diagram		RECORD 13.1: Describe in detail the selection of the persons included in the study (*i.e.,* study population selection) including filtering based on data quality, data availability and linkage. The selection of included persons can be described in the text and/or by means of the study flow diagram.	Methods: study sample section
Descriptive data	14	(a) Give characteristics of study participants (*e.g.*, demographic, clinical, social) and information			Results: Table 2. & 3.

		on exposures and potential confounders<br>(b) Indicate the number of participants with missing data for each variable of interest<br>(c) *Cohort study* - summarise follow-up time (*e.g.*, average and total amount)			
Outcome data	15	*Cohort study* - Report numbers of outcome events or summary measures over time<br>*Case-control study* - Report numbers in each exposure category, or summary measures of exposure<br>*Cross-sectional study* - Report numbers of outcome events or summary measures			Table 3.
Main results	16	(a) Give unadjusted estimates and, if applicable, confounder-adjusted estimates and their precision (e.g., 95% confidence interval). Make clear which confounders were adjusted for and why they were included<br>(b) Report category boundaries when continuous variables were categorized<br>(c) If relevant, consider translating estimates of relative risk into absolute risk for a meaningful time period			Table 4.
Other analyses	17	Report other analyses done—e.g., analyses of subgroups and interactions, and sensitivity analyses			Table 5a.<br>Table 5b.
**Discussion**					

For peer review only

Key results	18	Summarise key results with reference to study objectives			Discussion, paragraph 1, 6
Limitations	19	Discuss limitations of the study, taking into account sources of potential bias or imprecision. Discuss both direction and magnitude of any potential bias		RECORD 19.1: Discuss the implications of using data that were not created or collected to answer the specific research question(s). Include discussion of misclassification bias, unmeasured confounding, missing data, and changing eligibility over time, as they pertain to the study being reported.	Discussion, paragraph 5
Interpretation	20	Give a cautious overall interpretation of results considering objectives, limitations, multiplicity of analyses, results from similar studies, and other relevant evidence			Discussion, paragraph 2 -3
Generalisability	21	Discuss the generalisability (external validity) of the study results			Discussion, paragraph 2 -3
**Other Information**					
Funding	22	Give the source of funding and the role of the funders for the present study and, if applicable, for the original study on which the present article is based			Funding statement
Accessibility of protocol, raw data, and programming code		..		RECORD 22.1: Authors should provide information on how to access any supplemental information such as the study protocol, raw data, or programming code.	Data sharing statement

*Reference: Benchimol EI, Smeeth L, Guttmann A, Harron K, Moher D, Petersen I, Sørensen HT, von Elm E, Langan SM, the RECORD Working Committee. The REporting of studies Conducted using Observational Routinely-collected health Data (RECORD) Statement. *PLoS Medicine* 2015; in press.

*Checklist is protected under Creative Commons Attribution (CC BY) license.
